# Synaptic molecular imaging in spared and deprived columns of mouse barrel cortex with array tomography

**DOI:** 10.1038/sdata.2014.46

**Published:** 2014-12-23

**Authors:** Nicholas C Weiler, Forrest Collman, Joshua T Vogelstein, Randal Burns, Stephen J Smith

**Affiliations:** 1 Graduate Program in Neurosciences, Stanford University School of Medicine, Stanford, California 94305, USA; 2 Department of Molecular and Cellular Physiology, Stanford University School of Medicine, Stanford, California 94305, USA; 3 Allen Institute for Brain Science, Seattle, Washington 98103, USA; 4 Department of Statistical Science, Duke University, Durham, North Carolina 27708, USA; 5 Department of Biomedical Engineering, The Johns Hopkins University, Baltimore, Maryland 21218, USA; 6 Department of Computer Science, The Johns Hopkins University, Baltimore, Maryland 21218, USA

## Abstract

A major question in neuroscience is how diverse subsets of synaptic connections in neural circuits are affected by experience dependent plasticity to form the basis for behavioral learning and memory. Differences in protein expression patterns at individual synapses could constitute a key to understanding both synaptic diversity and the effects of plasticity at different synapse populations. Our approach to this question leverages the immunohistochemical multiplexing capability of array tomography (ATomo) and the columnar organization of mouse barrel cortex to create a dataset comprising high resolution volumetric images of spared and deprived cortical whisker barrels stained for over a dozen synaptic molecules each. These dataset has been made available through the Open Connectome Project for interactive online viewing, and may also be downloaded for offline analysis using web, Matlab, and other interfaces.

## Background & Summary

Many hundreds of distinct proteins are involved in the development of synapses and the mechanics of synaptic signaling^1–7^, and this complex molecular architecture exhibits striking diversity between brain regions, cell types, and even individual synapses belonging to the same neuron^8^. This complexity at first seems daunting, but may in fact enable researchers to better characterize synaptic populations and their experience-dependent modification. In particular, the definition of different synapse types based on characteristic expression of distinct molecular constituents by may enable identification of subpopulations involved in learning, while identifiable patterns of molecular modifications at existing connections may be used to reveal functional changes in synaptic strength^8^.

**Figure 1:**
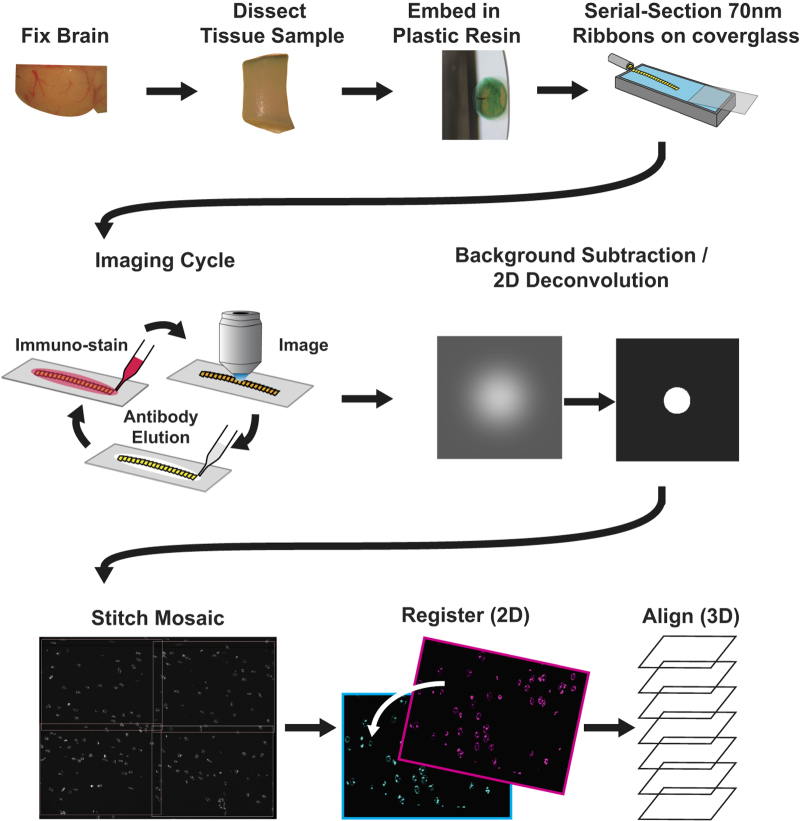
Pipeline of ATomo imaging and reconstruction. *Top Row: array production.* Fixed brain tissue is dissected into a small sample, in this case a tissue punch, and embedded in resin (generally LR White). This embedded sample is sectioned into ribbons of ultrathin (50–200 nm) serial sections, which are each affixed to a microscope coverglass to form a stable array. *Middle Row: imaging and image processing.* A ribbon array is stained with antibodies against selected antigens, and indirect immunofluorescence (IF) is imaged using a high-resolution objective. The antibodies can be removed from the ribbon using a high-pH elution solution, and the array can then be used again for multiple cycles of immunostaining and imaging. Image processing software improves the resolution of the resulting images. *Bottom Row: volume reconstruction.* Custom software is used for stitching, registration, and alignment of acquired images into volumetric reconstructions of the original tissue sample. ‘Mosaics’ comprising multiple microscope fields of view are stitched into individual images of the same location in each serial section across the ribbon. Images of each serial section from different imaging sessions (with different antibody stains applied) are then registered into the same 2-dimensional data space. Serial sections are then three-dimensionally aligned with one another across the ribbon. Software used for image processing and reconstruction can be found at http://smithlabsoftware.googlecode.com.

Array tomography (ATomo) is uniquely well suited to proteomic mapping of synaptic circuits. Ultrathin sectioning of resin-embedded tissue samples enables immunohistochemical multiplexing and high-resolution imaging of millions of synapses in situ, followed by computational reconstruction into precisely aligned image volumes^9,10^. The ability to measure the molecular composition of many individual synapses in the context of the larger circuits they comprise should greatly enhance our understanding of the roles of diverse synapse subsets in neuronal information storage and plasticity. (See Figure 1 for a summary of the method.)

**Figure 2:**
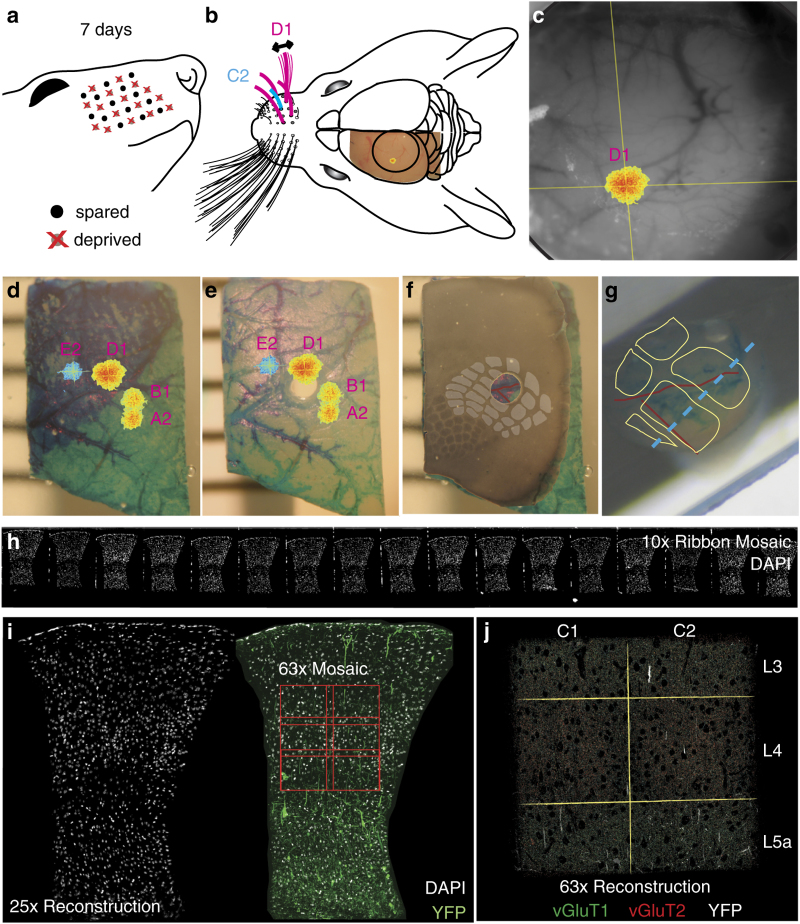
Functionally guided barrel column extraction. (**a**) Chessboard pattern of whisker deprivation. (**b**) Spared whiskers (magenta) surrounding the C2 whisker (cyan) were stimulated in anesthetized mice. Intrinsic optical signal (IOS) imaged transcranially over left somatosensory cortex (S1). (**c**) *In vivo* images of D1-whisker stimulation-evoked IOS peak (pseudocolor) and cerebral surface vasculature (grayscale). (**d**) Fixed and dissected left S1 cortex with tissue paint and registered IOS peaks (pseudocolor). (**e**) Remainder tissue after removal of tissue punch centered on the C2 whisker column. (**f**) Cytochrome oxidase (CO)-stained 80 micron-thick section of remainder tissue registered to the intact remainder tissue, with barrel field traced to confirm the correct punch localization (gray overlay). (**g**) Estimated barrel column positions within embedded tissue punch (yellow outlines) based on vascular and tissue paint features (cf. red traced blood vessels in panel f) with estimate of optimal cross-section through the C1 and C2 barrel columns parallel to the C-row axis (dashed cyan line). (**h**) Portion of ATomo ribbon imaged for DAPI at 10x magnification. (**i**) Maximum-intensity z-projection (MIP) of volume reconstruction of 25x magnification images of ribbon in h. Left: DAPI (gray). Right: DAPI (gray) and YFP (green). High-resolution imaging is targeted to C1 & C2, L3-L5a (red outlines). (**j**) MIP of multi-session volume reconstruction of 63x magnification images of region shown in (**i**), with YFP (grey) vGluT1 (green) and vGluT2 (red).

To enable this analysis, we have used ATomo to image the synaptic molecular architecture of neighboring whisker-associated columns of mouse somatosensory cortex (S1). The whisker region of S1 is organized as a grid-like spatial map, with rows of thalamically-innervated layer 4 (L4) ‘barrels’ corresponding to the layout of whiskers on the face^11,12^ (see Figure 2). The circuitry of this cortical barrel field is highly plastic, able to reorganize itself quickly if the whisker layout is altered^13^. The neurons immediately above and below a given barrel (the ‘barrel column’) normally respond primarily to stimulation of a single principal whisker (PW), but also exhibit weaker surround-whisker (SW) responses. Loss of input from the column’s PW depresses the column’s response to this whisker, while potentiating responses to spared SWs, which can come to dominate circuit activity in the deprived column^14,15^. This produces a remapping of the barrel field such that the representations of spared whiskers expand into neighboring deprived columns. Plasticity of many different components of the barrel column circuit have been implicated in this remapping, including excitatory and inhibitory synapses in thalamorecipient L4, superficial L2/3 and deep L5 (refs. 16, 17 and 18).

In our experiments, we trimmed alternating facial whiskers of adult mice in a ‘chessboard’ pattern, every 2-3 days for 7 days, which has been shown to produce significant functional and structural plasticity in barrel cortex well into adulthood^19–21^. We developed a method to precisely dissect pairs of neighboring barrel columns for ATomo imaging, and produced volume reconstructions of stains against many functionally important synaptic molecules in these adjacent spared and deprived columns. Additionally, this data contains cell-type specific transgenic labeling of layer 5 pyramidal neurons with YFP^22,23^, as well as immunohistochemical labeling of PV+ interneurons^24–26^. Thus, synaptic subpopulations may be classified based on characteristic molecular signatures or association with YFP+ pyramidal neurons or PV+ interneurons, and the prevalence of these populations as well as changes in expression of molecules thought to play a role in experience-dependent plasticity may be compared between spared and deprived columns.

The Chessboard Dataset described here comprises over 6 million cubic microns of neocortical tissue within a well-defined microanatomical structure, stained for over a dozen different synaptic proteins and imaged at synaptic resolution. As such, it is likely to be a valuable resource for researchers interested in statistical characterization of the diversity of protein expression patterns in mouse neocortical synapses and the effects of experience-dependent plasticity on these patterns^8^. We make this dataset publicly available alongside two supplementary datasets (see Data Records) in the hopes that other researchers will apply their own creative approaches to discovering patterns of synapse molecular diversity and novel signatures of experience dependent cortical learning.

## Methods

### Animals

All procedures related to the care and treatment of animals were approved by the Administrative Panel on Laboratory Animal Care at Stanford University. Animals used to generate these datasets were derived from hemizygous transgenic founder mice of the thy1-yfp H line^22^ purchased from Jackson Labs (Strain Name: B6.Cg-Tg(Thy1-YFP)HJrs/J, Stock number: 003782). Mouse Ex1 was excluded from the Chessboard Dataset (see Data Records) because it was found not to express YFP. Thereafter, we used the Transnetyx genotyping service to restrict experiments to YFP-positive individuals based on the results of automated rtPCR comparison with JAX gene sequences.

### Chessboard whisker deprivation

We induced experience dependent map plasticity in 8 adult male mice (P63-70) by unilaterally trimming facial whiskers on the right side of the face in a chessboard pattern (that is, alternating spared and trimmed whiskers) every 2-3 days for 7 days (Figure 2a). We trimmed whiskers to within 1 mm of the face, and did not observe significant regrowth between trimming sessions. Of the 7 mice included in the Chessboard Dataset (see Data Records), 4 had the chessboard pattern centered on a spared C2 whisker (C2 spared), and 3 had the chessboard pattern centered on a deprived C2 whisker (C2 deprived). Animals were group-housed and supplied with cardboard tubes for mild enrichment and to discourage whisker barbering by cagemates^27^. Mice were excluded from these dataset if any whisker loss was observed in addition to the chessboard trimming.

### Rodent surgery

Following 7 days of chessboard deprivation, we imaged whisker-evoked intrinsic optical signals (IOS)^28^ to localize barrel columns C1 and C2 for dissection. Mice were sedated with intraperitoneal injections of 0.05 ml chlorprothixene and anesthetized with isoflurane (0.8% in O_2_). Mice were kept on a heating pad, and we ensured that mice remained at a stable level of anesthesia by monitoring their core temperature and tracking their respiration rate. We also locally anesthetized the scalp with subcutaneous injections of ~0.01 ml lidocaine. We covered the eyes with petroleum jelly and provided hydration with a pre-operative subcutaneous injection of 0.5 ml physiological saline.

We stabilized the heads of the mice with ear-bars and surgically exposed a region of the skull overlying the left somatosensory cortex. We then affixed a custom metal head-plate with a 2 mm round imaging window to the exposed skull approximately centered on the location of left barrel cortex, and clamped this head plate to a metal post attached to the imaging apparatus. Once the mice were thus head-fixed, we removed the ear-bars, filled the imaging window with warm low-melting-point agarose, and covered the window with a round glass coverslip.

### Intrinsic Optical Signal (IOS) imaging

To identify the location of barrel-columns C1 and C2 without producing additional plasticity in these columns by direct stimulation, we used IOS imaging to map the representations of adjacent spared whiskers (Figure 2b). In C2-spared mice, we mapped the locations of the spared A2, B1, D1 and E2 whisker representations, while in C1-spared mice, we mapped the spared A1, B2, D2, and E1 whiskers. We used a custom piezo-electric actuator (PiezoSystems) to stimulate individual right facial whiskers at 10 Hz in 5-second bouts with an inter-stimulus interval of 15 s. The imaging window was illuminated with a 630 nm red/orange LED light (LumiLEDs), and a Pantera 1M30 camera (Dalsa) equipped with a nose-to-nose macro-lens was used to record changes in reflectance through the skull^29^. We measured IOS with the camera focused 500 microns beneath the cortical surface to maximize signals originating L4 barrels.

IOS peaks associated with the 0.05 Hz period of whisker stimulation bouts were obtained using LabView software designed by David Ferster, which calculated the real-time fast fourier transform (FFT) of changes in reflectance at this frequency. This FFT-based method allows fast and robust detection of stimulus-associated IOS peaks with little interference from other cardiovascular rhythms, which occur at much higher frequencies^30^. Clear IOS peaks were generally detected within 15 bouts (~5 min) of stimulation. For each whisker, once an IOS peak was established, we photographed the vascular pattern at the surface of the cortex with contrast enhanced by 530 nm green LED illumination (LumiLEDs). Because the position of the camera relative to the imaging window could shift slightly as we switched whiskers, we registered the vascular images and their paired IOS peaks to map the location of each whisker-associated peak relative to the surface vasculature (Figure 2c).

### Barrel column dissection

Following IOS imaging, animals were deeply anesthetized with 5% isoflurane, their brains extracted and the left somatosensory cortex dissected and immersed in fixative (4% PFA, 2.5% sucrose in 0.01 M PBS)^31^. We estimated the locations of the C-row barrel columns by registering *in vivo* images of the surface vasculature collected under 530 nm illumination to the visible vasculature of the fixed somatosensory cortex tissue. We used tissue paint (Polysciences) to help reveal additional vascular features in the fixed tissue and to highlight the estimated position of the C-row of the barrel field (Figure 2d). We embedded this painted tissue in agarose gel for stability, and used a custom guide-chamber and sharpened oval tube (inner diameter ~1×0.75 mm) to precisely target extraction of a tissue punch centered on the border between barrels C1 and C2 (Figure 2e), as estimated by vascular and tissue-paint features registered to the functionally measured ISI peaks. The punched tissue was then embedded in LR-white resin for ultra-thin sectioning into ATomo ribbons (see ATomo Tissue Processing, below).

### Cytochrome oxidase staining

We confirmed that our punch contained the C1 and C2 barrel columns by making 80-micron sections of the somatosensory cortex tissue remaining after the tissue punch procedure (hereafter ‘remainder tissue’), and stained these for CO to reveal the pattern of the barrel field in layer 4. We then registered our standard barrel field map to the CO stain of remainder tissue sections to reveal which barrels overlapped with the punched-out hole in these sections (Figure 2f).

The CO-stained sections also allowed us to estimate the precise position and orientation of the columns contained within the embedded tissue-punch in order to plan the sectioning of ribbons for ATomo (Figure 2g). For this purpose, we registered images of the vascular features and tissue paint at the pia surface at each stage of the dissection procedure. Based on these common features, we were able to register images of 1) the fixed tissue prior to punch extraction, 2) the superficial sections of post-punch remainder tissue, and 3) the surface of the punched tissue before and after embedding. We aligned adjacent sections of the remainder tissue from the superficial sections containing vascular features down to the L4 sections containing the CO-stained barrel field based on vertically projecting capillaries visible from section to section. Following this alignment, the standard barrel field map and embedded punch images were also in registry, and so we could estimate the locations of the embedded barrel columns with great precision.

### ATomo tissue processing

We dehydrated the tissue punches containing C1 and C2 columns in ascending alcohols up to 80% to retain YFP fluorescence, and embedded them in LR White resin according to previously published methods^9,31,32^.

The resin block was oriented based on the registration procedure described above (Figure 2g) and sectioned into ribbons of 70 nm serial sections (~50 sections/ribbon) parallel to the axis of the C-row such that each ribbon comprised ~3.5 micron cross-sections of the embedded C1 and C2 columns and contained all layers of cortex between the pia surface and the subcortical white matter^33^. We collected 40–100 ribbons from each embedded block, enough to contain all or most of the targeted pairs of barrel columns. Although we could in principle attempt to reconstruct the columns in their entirety, the scale of such a project was outside the capabilities of our current image acquisition and reconstruction pipeline. Instead, for each block we identified the ribbon that best approximated a plane passing through the centers of both neighboring barrels for imaging and reconstruction (Figure 2g). To aid in this selection, we took sequential pictures of the surface of the punch during sectioning to keep track of our progress through the columns, so that we could select ribbons from as near as possible to the C-row center (that is, equidistant from rows B and D).

Note that for most animals, ribbons were cut from the tissue punch starting from the E-side of the C-row (Ex1, Ex2, Ex3, Ex6, Ex10, Ex14) while others were cut from the B-side (Ex12, Ex13), producing different left-right orientations of the columns in the final data volumes (See Table 1).

### ATomo ribbon imaging

For all imaging experiments, we first create a low-resolution overview of the ribbon to be imaged by making a mosaic image at low magnification (10x) of a DAPI stain for cell nuclei included in the mounting medium (molecular probes ref S36939) (Figure 2h). To ensure that we selected good quality ribbons for high-resolution immunohistochemical imaging, we imaged DAPI and intrinsic YFP fluorescence in several ribbons surrounding the estimated row center with a 25x magnification objective (Zeiss). These 25x images allowed us to reconstruct large cross-sections of barrel columns (Figure 2i; see *ATomo Image Processing and Reconstruction*, below), which aided in selecting ribbons taken from closest to the barrel centers, based on their larger barrel hollows visible as lower DAPI and YFP density. Once a ribbon was selected, the 25x DAPI/YFP images were also used to identify boundaries between columns and layers to guide higher magnification imaging of layers 3–5a in columns C1 and C2. Column boundaries could be detected by the higher density of YFP dendrites running between columns. The centers of L4 barrels were identified by their low DAPI density compared to higher density ‘septal’ regions between barrels, which is a characteristic feature of mouse barrel cortex^15^.

For selected ribbons from each chessboard-deprived animal, we performed 6–8 rounds of triple or quadruple immunostaining, paired with high-resolution imaging with a 63x plan-apochromat objective (Zeiss)^34,35^. For all sections in each ribbon, we imaged a region of the section corresponding to layers 3-5a in all of the columns present (C1-C2 in 5 animals, C1-C3 in 1 animal). Automated mosaic imaging of the selected region was targeted using custom software (MosaicPlanner; available at *https://smithlabsoftware.googlecode.com*/). For several ribbons, to minimize focus artifacts resulting from deviations of the 70 nm sections from the objective’s limited focal plane, z-stacks of each mosaic position were imaged and then computationally merged using an extended depth of field algorithm (Zeiss Axiovision).

### ATomo image processing and reconstruction

*All custom code described in this section is available for download at* *https://smithlabsoftware.googlecode.com* .

#### Image processing

Following imaging, we used plugins available in the FIJI image processing software as well as custom routines to create high-dimensional volume reconstructions of the imaged tissue with all of the antibody stains in registry. First, all images were background subtracted using a 20 pixel rolling ball filter (FIJI/ImageJ) and deconvolved (Matlab) based on empirical point-spread functions measured with fluorescent tetraspeck beads. Following deconvolution, the effective resolution of the images was 100×100×70 nm (ref. 36).

#### Mosaic stitching

We used a DAPI nuclear stain present in our mounting medium in all imaging sessions as a fiducial marker both for stitching together adjacent microscope fields of view and registering images of the same tissue sections taken in different imaging sessions (see below).

#### 2D registration

Minor shifts in the position of the coverslip-affixed ribbon between imaging sessions can be simply modeled as rigid linear transformations to achieve appropriate session registration using custom FIJI/ImageJ plugins. These transformations were calculated based on SIFT features detected in the corresponding DAPI images from each imaging session^37^. These features were filtered using RANSAC^38^ to detect inliers specifying the model for rigid transformation between sessions, which could then be applied to images from the other channels of that session to bring them into registry with the first session.

#### 3D alignment

Unlike the rigid transformations used for 2d registration between imaging sessions, mechanical deformations of the tissue during the process of sectioning make the transformations needed for 3d alignment of ultrathin serial-section images significantly nonlinear. Synapse-scale alignment was achieved with another custom FIJI/ImageJ plugin by applying the elastic algorithm developed by Saalfeld and colleagues to the Synapsin1 channel^39^. This channel was used for fine alignment because it densely and reliably stains synapses across multiple 70 nm sections. The alignment calculated from Synapsin1 was then applied to all of the other channels already in registry to produce full multi-channel reconstructed data volumes.

## Data Records

There are 3 data records associated with this data descriptor, all derived from serial-section ribbons from 9 chessboard-deprived mice (Table 1), imaged across multiple rounds of staining with a panel of 21 antibodies (Table 2).

### 1: Chessboard dataset

The primary Chessboard Dataset consists of 12 reconstructed data volumes (Table 3 (available online only)) comprising 20–25 immunohistochemical channels each (Table 4 (available online only)). These data volumes were reconstructed from multiple rounds of imaging of eight ribbons of serial sections containing paired spared and deprived barrel columns from seven chessboard-deprived mice (Table 5 (available online only)).

The Chessboard Dataset is hosted for interactive browsing and download through the Open Connectome Project (OCP) at http://openconnecto.me. (For further instructions, see Data Access, below, as well as Usage Notes: OCP Data Access.) Individual data files for each channel in each volume reconstruction are also available through FigShare (Data Citation 1), as files 8–295 described in Supplementary Table 1.

#### Chessboard dataset caveats

Please note that the number of animals, imaged ribbons, and derived data volumes differ because (1) two ribbons from mouse Ex12 were imaged and (2) multiple barrel columns from ribbons Ex2R18, Ex3R43, and Ex6R15 were reconstructed separately into 2-3 columnar data volumes per ribbon. Also, note that animal ribbon Ex14-R58 was subject to several significant problems during antibody staining (see Table 5 (available online only)), particularly as relates to the staining for vGluT2. We include the derived data volumes in the present dataset with this caveat, and we have excluded this ribbon from the quantification of vGluT puncta density (See Usage Notes: Analysis Recommendations & Caveats).

#### Data access

There are two ways to access the Chessboard Dataset through OCP: 1) interactively browsing through entire data volumes over the web through OCP’s CATMAID interface, which is ideal for quick visual inspection of the data, or 2) by downloading specific data regions (called ‘cutouts’) through OCP’s HDF5 interface, which is ideal for handling the data for analysis. OCP also has its own API for those wishing to interact more deeply with the project’s data structures. For the most up-to-date descriptions of OCP’s services, visit http://openconnecto.me/#!services/chru.

#### Online data viewing

CATMAID stands for Collaborative Annotation Toolkit for Massive Amounts of Image Data (http://catmaid.org/). OCP uses this interface to enable fast, browsable online data viewing as well as collaborative data annotation. To view the data, visit http://openconnecto.me/catmaid and browse projects labeled ‘Array Tomography (Weiler *et al.*)’. You may view these projects only by selecting Home> Array Tomography in the upper left-hand corner of the browser window. We have curated several useful multi-channel volumes to explore, but users may also construct their own combinations of channels to visualize different molecular relationships using hdf5 data cutouts (see below).

#### Viewing/downloading data cutouts

To access data volumes for analysis, OCP provides a ‘cutout service’ which allows users to download arbitrary 3d data cubes using the HDF5 interface through Matlab, R, C, C++, or C# or by downloading the NumPy pickle for use with python. (See Usage Notes: OCP Data Access for a guide to using the hdf5 web interface to view or download cutouts.).

For additional documentation of the cutout service, see the OCP wiki page at the following address: http://tinyurl.com/OCP-cutout-wiki. We have prepared sample scripts for data access using MATLAB at https://gist.github.com/ncweiler. The cutout service also enables collaborative annotation of the data (labeling neuronal or synaptic structures, for instance). These metadata annotations are registered to the original data and can be either used privately or shared publicly. See more details on data annotations at http://tinyurl.com/OCP-annotation-wiki.

### 2: Chessboard dataset low-magnification overview images

Prior to the high-resolution, high-magnification imaging that generated the Chessboard Dataset image volumes, we first imaged intrinsic YFP and DAPI-stained cell bodies in each ribbon at low magnification. These images allowed us to create a roughly aligned reconstruction of the complete ribbons, which we then used to help identify anatomical layers and columns for the subsequent high-magnification imaging that generated the Chessboard Dataset image volumes. 2d projections of these whole-ribbon reconstructions, created using either a standard deviation projection or maximum intensity projection (FIJI), are also made available through FigShare as RGB tiff images to support the identification and analysis of specific anatomical regions (Data Citation 2).

### 3: Elution-test dataset

The supplementary Elution-Test Dataset consists of data volumes derived from seven short ribbon segments derived from mouse Ex1 (Table 6 (available online only)), each stained and imaged repeatedly with a different panel of antibodies (Tables 7 and 8 (available online only)). Analysis of stain repeatability can help estimate the signal to noise ratio of each antibody—the likelihood that a punctum of stain will be seen on repeated staining of the same tissue (see Technical Validation).

In the Elution-Test Dataset, each ribbon was divided into distinct segments, which were stained separately with different antibody panels. These ribbon segments are designated with the letters A through F. Unlike the Chessboard Dataset, only small fields of view from layer 4 were imaged in each of these ribbons, since the objective of this experiment was antibody testing, rather than analysis of differences between regions. Six of ribbon segments have been reconstructed into data volumes, but one other ribbon segment (Ex1-R02A) was imaged at four non-consecutive tissue sections, preventing volumetric reconstruction.

The Elution-Test Dataset may be downloaded from Figshare (Data Citation 1), as files 1–7 described in Supplementary Table 1. Unlike the Chessboard Dataset, each of these files is a zip file containing all channels for a given data volume.

### OCP—FigShare dataset cross-reference

See Supplementary Table 1 to cross-reference data files available through the Open Connectome Project (OCP) and FigShare. Files comprising the Chessboard and Elute-Test Datasets (Data Citation 1) are listed alongside the animal and ribbon they derive from, the antibody stains they include, and the accession addresses for the data at both OCP and FigShare.

Note that the OCP web addresses as written will display the full extent of the first slice (z-section) of a multi-slice volume image. To view a smaller region in x or y, adjust the values in the second-to-last and third-to last terms in the HDF5 web address. To view a different slice, adjust the final term in the HDF5 address. (For additional instructions regarding the OCP web interface, see Usage Notes: OCP Data Access.)

## Technical Validation

### Antibody specificity

All antibodies used in this study have been validated by their originators as reliable and specific to the antigens of interest, and tested within our laboratory for reliable and repeatable staining of ATomo ribbons of LR-white embedded tissue sections. We make sure all antibodies stain in patterns that correspond to their expected synaptic localization. That is, for example, each GABAergic antibody was screened to confirm that it co-stained a significant number of putative GABAergic synapses defined by multiple other GABAergic antibodies, and that it did not excessively stain within cell bodies or blood vessels, where synapses would not be expected.

We have quantified these expected staining patterns by performing a pairwise 2-dimensional cross-correlation analysis to quantify spatial correlations (colocalization) between the principal antibodies used in these experiments. Pairwise cross-correlation plots were generated by shifting pairs of registered antibody channels against each other in x and y and calculating 2d image correlations for each spatial shift.

**Figure 3:**
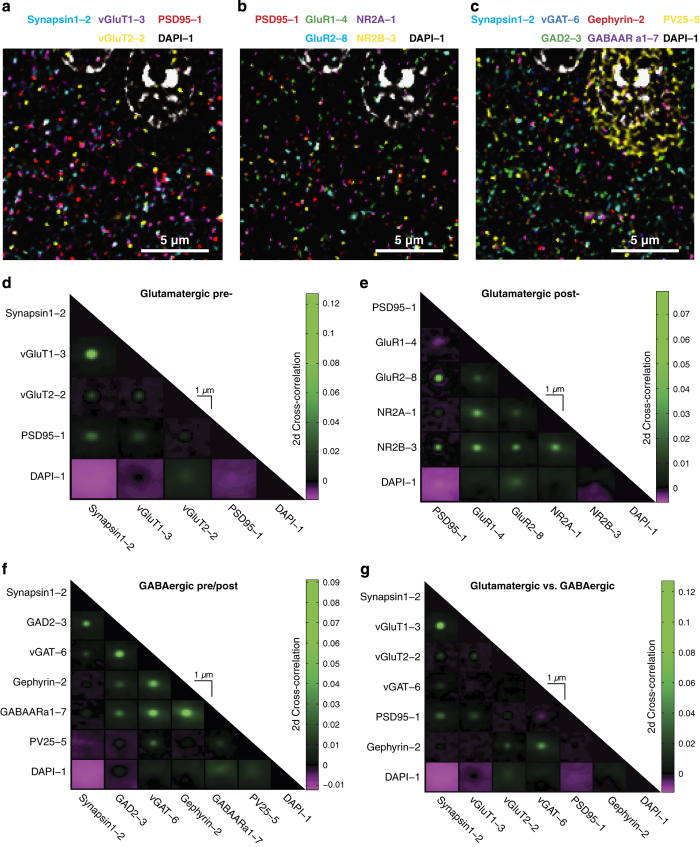
Validation of synaptic antibody colocalization. (**a**–**c**) Multi-channel composite images of related synaptic protein stains in a subregion of a single serial section of the Ex10-R55 ribbon. *Scale bar 5 um.* (**d**–**g**) 2-dimensional cross-correlation analysis of the colocalization of antibody stains specific to the pre- and postsynaptic compartments of glutamatergic and GABAergic synapses. Each plot contains an array of panels representing correlation between different stains of a subregion of the Ex10-R55 ribbon as a function of spatial shifts in x and y (shifts up to 10 px shown), averaged over stacks of 10 serial sections. (**a**,**d**) Colocalization of stains for presynaptic glutamatergic proteins (Synapsin1, vGluT1 and vGluT2) and comparison with postsynaptic PSD95 and nuclear DAPI stains. (**b**,**e**) Colocalization of stains for postsynaptic glutamatergic proteins (PSD95, GluR1, GluR2, NR2A and NR2B) and comparison to nuclear DAPI stain. (**c**,**f**) Colocalization of stains for presynaptic (Synapsin1, vGAT, GAD, and PV25) and postsynaptic (GABAARa1 and Gephyrin) GABAergic proteins and comparison with nuclear DAPI stain. (**g**) Colocalization of glutamatergic and GABAergic vesicular neurotransmitter transporters (vGluT1, vGluT2, vGAT) relative to presynaptic marker Synapsin1, postsynaptic markers PSD95 (glutamatergic) and gephyrin (GABAergic), and DAPI-stained cell nuclei.

These pairwise cross-correlations demonstrated sharp central peaks for colocalized antibodies, and broader noisier values for poorly colocalized antibodies. Examining the cross-correlations at small 2d shifts between images reveals that pairs of antibodies which are expected to colocalize within either pre- or postsynaptic compartments (for example, Synapsin1 and vGluT1 or PSD95 and GluR2, respectively) have sharp peaks of correlation, while pairs of antibodies which represent associated pre- and postsynaptic compartments (for example, Synapsin1 and PSD95) have broader, more diffuse cross-correlation peaks (Figure 3).

To quantify these correlations, we measured the absolute peak of the cross-correlation, and calculated a half-maximum value based on the average background correlation. Using Matlab, we also calculated a 2d contour of the cross-correlation peak at the half-maximum value, and measured the area of this contour and calculated the diameter of a circular contour with the same area. These values are displayed in Table 9.

### Antibody consistency

In previously published work, we demonstrated that multiple rounds of elution have minimal effect on tissue antigenicity as quantified by the consistency of staining with an antibody against Synapsin1 across multiple rounds of imaging^9^. In the present datasets, we also observed comparable staining quality irrespective of the multiple rounds of stripping and staining.

**Figure 4:**
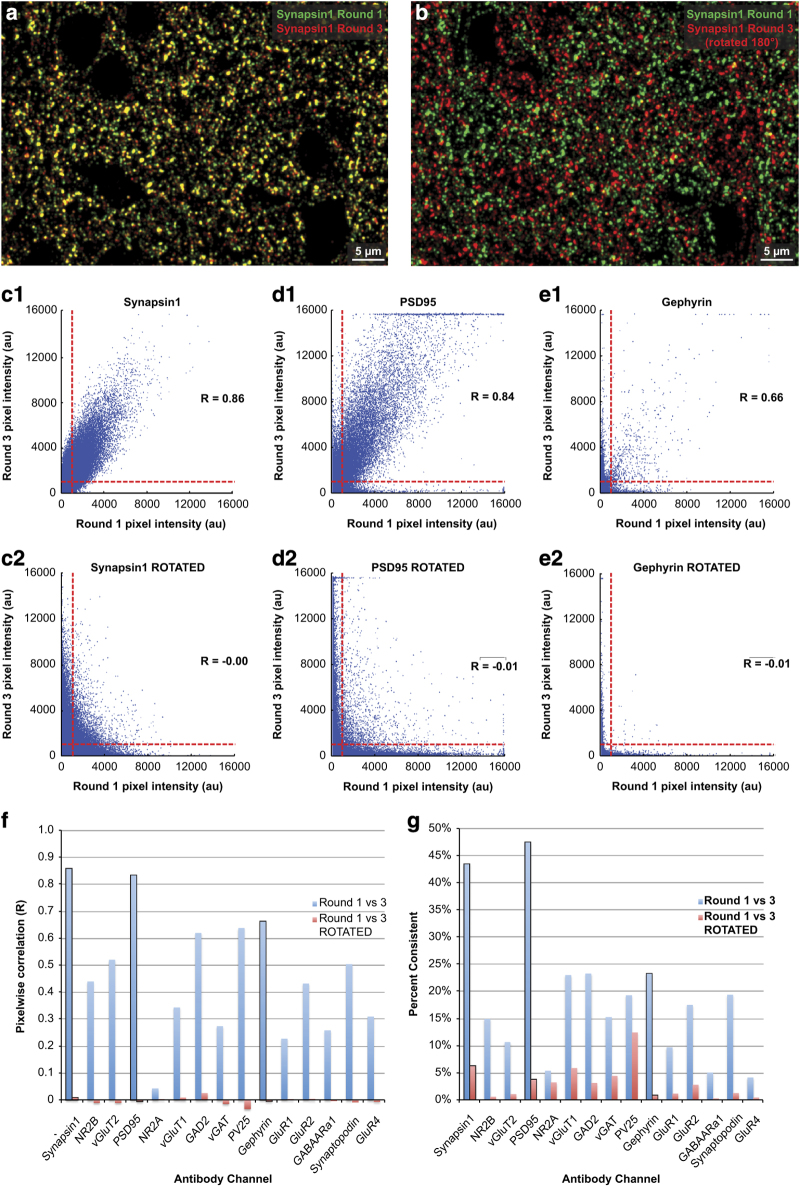
Evaluation of stain robustness across imaging sessions. (**a**) False-color composite image comparing the first stain of a subregion of ribbon Ex1-R02A with an antibody against Synapsin1 protein (green) with the third sequential stain of the same region (red). Areas of overlap (yellow) represent consistent patterns of staining despite intervening rounds of stripping and restaining. (**b**) Here the image of the third round of staining has been rotated 180° to illustrate that the chance occurrence of overlapping staining is quite low, despite the high stain density in the two images. (**c1**) Synapsin1 immunofluorescence intensity at individual pixels compared between rounds 1 and 3. The R value represents Pearson’s correlation coefficient between the two images. An approximate threshold between foreground and background pixels (set at 1,000 a.u.) in each image is illustrated by horizontal and vertical red dashed lines. (**c2**) The same analysis with the image of round 3 rotated 180° to serve as a control for random colocalizations. (**d**,**e**) The same analyses for PSD95 and Gephyrin staining. (**f**) Comparison of correlation coeffients (R) for images of the first and third round of staining for 15 different antibodies. (**g**) For the same set of antibody stains, comparison of the ‘percent consistency’: the percent of pixels that were bright (above threshold) in either imaging session which were also bright in the other imaging session. See Table 10 for a full list of these values.

To further test the consistency of our staining procedure, we have performed three rounds of repeated staining with fifteen key antibodies on ribbons equivalent to those used to produce the Chessboard Dataset. We used 2d image correlation to assess the similarity of staining between the first and third rounds of imaging for each antibody (Figure 4a,c1,e1,g1). To compare the observed level of correlation to a proxy for ‘random’ colocalization, we rotated the images of the third imaging session 180° and repeated the analysis (Figure 4b,c2,e2,g2).

Since the large proportion of dark background pixels could be contributing significantly to these image correlations, we also computed a ‘percent consistent’ metric that measured the proportion of bright pixels that were bright in both images. We set an approximate threshold between foreground and background at 1,000 a.u., and quantified the proportion of pixels above this threshold in either imaging session that were also above threshold in the other imaging session. As with the correlation metric, we compared this ‘percent consistent’ metric between the observed colocalization and the ‘random’ colocalization achieved by rotating one image 180° (Figure 4g). This pixelwise metric is only a rough quantification of antibody consistency, but it provides a simple supplement to the correlation values described above.

We find that the antibodies against Synapsin1 and PSD95 produce highly consistent staining between sessions, while the rest (with the exception of NR2A) ranged from relatively good to relatively poor consistency, when compared with the extremely low values expected by random colocalization (Figure 4f,g, Table 10). This analysis corresponds well with previous unpublished observations by our lab, and suggests that each antibody channel represents a mixture of robust signal and stochastic variability, with a signal-to-noise ratio that varies between antibodies.

An important result of this analysis was that the NR2A antibody appeared to be completely unreliable, performing much more poorly than expected compared to random colocalization. Previous observations by our lab (data unpublished) and the correlation analysis presented in Figure 3 demonstrate a reasonable level of colocalization of this antibody with PSD95 and other glutamatergic post-synaptic markers. However, given the results of this analysis, we must recommend extreme caution in any interpretation of this antibody channel within the present dataset.

The stochasticity observed in some antibodies will be an important consideration in any analysis of the Chessboard Dataset. We do not yet have an adequate biophysical model to explain the source of this variability, which is why we have made this Elution-Test dataset available through Figshare for download and further analysis (Data Citation 1, Files 1–7 Supplementary Table 1; see Data Records). Fortunately, the component of stochasticity we observe in some stains is not as concerning as it might be if one hoped to use these antibodies on their own for synapse identification. ATomo presents the opportunity to use over a dozen antibodies in parallel to define synapses based on the convergence of multiple lines of immunohistochemical evidence. Therefore even a stain with low signal-to-noise can be useful in characterizing synapse diversity in combination with other, more reliable stains. For further discussion of recommended approaches to proteomic analysis of synapses using the Chessboard Dataset, please see Usage Notes, below.

## Usage Notes

### OCP data access

The HDF5 web interface enables anyone with an internet connection to access arbitrary data cutouts through their web browser or to download data cutouts using python or Matlab scripts. Several usage examples are listed below, followed by an explanation of the meanings of the HDF5 arguments used.

#### Data access examples

*Single-Channel 2d Web-View (8 bit grayscale)*

Template: *http://openconnecto.me/ocp/ca/* *[data token]/[orthogonal-view]/[channel token]/[resolution]/[x-range]/[y-range]/[z-range]/*

Example: *http://openconnecto.me/ocp/ca/Ex6R15C2/xy/DAPI-1/0/700,1700/500,1500/15/*

Result: *Produces cutout of DAPI-1 channel from data volume Ex6R15C2 at full resolution (‘0’) for x-range 0*–*1400, y-range 0*–*700, and z-slice 15.*

*Multi-Channel 2d Web-View (CYMRGB false color)*

Template: *http://openconnecto.me/ocp/ca/[data token]/mcfc/[orthogonal-view]/[channel tokens]/[resolution]/[x-range]/[y-range]/[z-range]/*

Example: *http://openconnecto.me/ocp/ca/Ex6R15C2/mcfc/xy/0,0,0,Synapsin1-2,PSD95-1,DAPI-1/0/700,1700/500,1500/20/*

Result: *Produces image of Synapsin1-2 (red), PSD95-1 (green), and DAPI-1 (blue) channels from data volume Ex6R15C2 at resolution 0 for x-range 0*–*1400, y-range 0*–*1400, and z-slice 20.*

Note: *Channels are displayed as Cyan, Yellow, Magenta, Red, Green, Blue in order listed. Use placeholder channel token ‘0’ as above to skip colors.*

*Multi-Channel 2d Matlab Download (CYMRGB false-color png file)*

Script: *https://gist.github.com/ncweiler/8075665*

Result: *This script downloads a specified 2d data cutout as a false-color png file. It also creates a data object in Matlab containing an RGB image of the cutout and displays the data as a false-color image. Channel colors are determined as in CYMRGB multi-channel false color web view above.*

*Multi-channel Volume Matlab Download (hdf5 data file)*

Script: *https://gist.github.com/ncweiler/7953445*

Result: *This script downloads a specified multichannel 3d data cutout as an hdf5 file. It creates a data object in Matlab containing one image stack for each channel. A user can then display specified slices of each channel stack.*

#### HDF5 arguments

As in the above examples, the following arguments will be needed to access data cutouts through the HDF5 interface:

*Data token*

To create a data cutout from a particular data volume, you must use the associated data volume token. For example, the token ‘Ex10R55’ would be used to access the volume reconstruction of that ribbon.

*Channel Token(s)*

Desired protein channels may be specified either by name or by numeric channel token (1–29, though NB that not all data volumes have 29 channels, and tokens may refer to different channels in different data volumes (See Tables 4 and 7 (available online only)).

*Resolution*

Data cutouts may be viewed in original or down-sampled resolution. ‘0’ specifies original resolution, and ‘1’ specifies half resolution.

*Orthogonal view*

The plane of view for 2d cutouts may be ‘xy’, ‘xz’, or ‘yz’.

*X-, Y-, and Z-Range*

These arguments specify the 2d or 3d extent of the data cutout. Use of the range arguments will depend on whether the cutout is a 2d plane or 3d volume. If accessing a 3d volume, a user must specify min and max values for all three axes, which set the height, width, and depth of the 3d cutout. In contrast, if accessing an xy plane, a user would specify min and max values for the x and y axes to determine the width and height of the 2d cutout, but would specify a single value for z to indicate the desired z-axis cross-section to be accessed.

#### Token/argument reference

A list of available public data tokens is available here: http://openconnecto.me/ocp/ocpca/public_tokens/. Available channel tokens, resolutions, and x-, y-, and z-ranges for each data volume may be viewed using the following url template: *http://openconnecto.me/ocp/ocpca/[data token]/info*/ (EG: http://openconnecto.me/ocp/ocpca/Ex10R55/info/). Alternatively, see Tables 3, 4, 6 and 7 (available online only).

### Choice of antibodies to screen for plasticity among diverse synapse subpopulations

**Figure 5:**
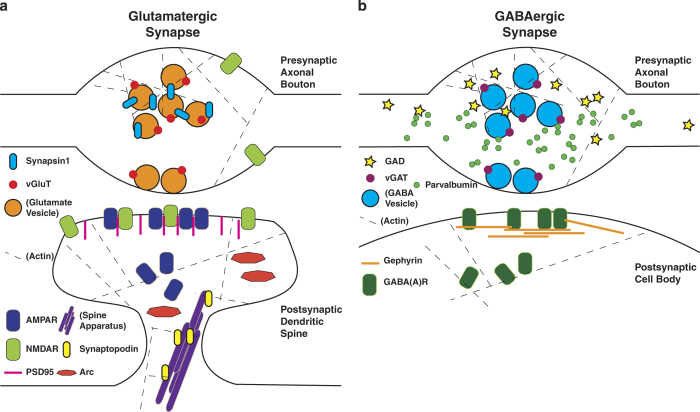
Molecular architecture of glutamatergic and GABAergic synapses. This figure presents the synaptic organization of the molecules used in the above experiments in cartoon form for glutamatergic (**a**) and GABAergic (**b**) synapses. Presynaptic axonal boutons are at the top for both synapses classes, and postsynaptic targets (here a dendritic spine and the surface of a cell body, as is typical of glutamatergic and GABAergic synapses, respectively). Molecules or structures not explicitly stained for in the above experiments are labeled with parentheses. Please see Usage Notes: *Choice of Antibodies to Screen for Plasticity among Diverse Synapse Subpopulations*, above, for further descriptions and references relevant to this figure.

ATomo’s ability to sequentially stain multiple different synaptic proteins and image their localization at high resolution presents the opportunity to define synapses by the spatial colocalization of multiple synaptic molecular constituents, whereas measurements of single antibodies could be noisy or misleading. See Figure 5 for a summary of the subsynaptic localizations of the antigens stained against in these experiments.

### Markers of glutamatergic synapses

**Synapsin1** is a vesicle-associated actin-binding protein. Staining for Synapsin1 is found at the majority of glutamatergic and GABAergic synapses in cortex, although we have observed it to be brighter at glutamatergic synapses^10^. This highly robust stain (see Figure 4) is a useful proxy for identification of loci of putative synapses, which can then be subject to additional classification and molecular characterization^10,40^. Expression of distinct isoforms of the vesicular glutamate transporter (vGluTs) has commonly been used to distinguish between axonal boutons of **vGluT1**-expressing intracortical synapses and **vGluT2**-expressing thalamocortical synapses. Some presynaptic terminals stain for both vGluT1 and vGluT2, which are likely to originate from a subpopulation of thalamocortical neurons expressing vGluT1 in addition to the typical thalamically expressed vGluT2 (refs 41,^42^), though others have reported vGluT2 expression in normally vGluT1-expressing intracortical synapses^43^.

**PSD95** is postsynaptic scaffolding molecule specific to glutamatergic synapses^44–46^. It is located close to the post-synaptic plasma membrane, and can thus also be used to define the post-synaptic compartment of the synapse adjacent to a Synapsin1-stained presynaptic compartment. Antibodies against glutamate receptor subunits including **GluR1**, **GluR2**, **NR2A**, and **NR2B** also stain the postsynaptic membrane of glutamatergic synapses^47–49^, and are therefore expected to colocalize with PSD95 (Figure 3). Different components of cortical microcircuits are also thought to exhibit characteristic differences in NR2 subunit expression^48,50^. However, a considerable literature describes additional presynaptic and extrasynaptic expression of these molecules^51–55^, suggesting that these stains should primarily be used in combination with other synaptic markers such as PSD95 and Synapsin1 to characterize receptor expression in different synaptic compartments.

### Markers for GABAergic synapses

Antibodies against glutamate decarboxylase (**GAD**), an enzyme responsible for synthesizing the neurotransmitter GABA, and **vGAT**, a vesicular GABA transporter, both stain presynaptic terminals of inhibitory synapses and can be used to differentiate these from excitatory synapses^56–58^. vGAT protein is thought to be translated locally at GABAergic and glycinergic boutons, making this stain highly specific for inhibitory synaptic (as opposed to extrasynaptic) loci^58^.

GABAergic synapses express a distinct postsynaptic scaffolding protein, **gephyrin**, which can be used to identify these inhibitory synapses from PSD95-expressing glutamatergic synapses^59–61^. As with glutamatergic synapses, stains for postsynaptic GABA receptor subunits can be used to further identify and characterize these postsynaptic compartments. A multitude of GABA receptor subunits are expressed in the brain^61^. One common subunit, **GABA(A)Ra1,** has been stained for in these experiments. This subunit is present in one of the largest subclasses of GABA(A) receptors and so can be used in combination with **gephyrin** staining to identify and subclassify the postsynaptic elements of GABAergic synapses^62^.

**Parvalbumin (PV)** is a calcium binding protein expressed by a large subclass of GABAergic neurons, including fast-spiking basket cells and chandelier cells. This stain can therefore be used to identify presynaptic GABAergic terminals belonging to these cell classes^26,63^. PV+ basket cell and chandelier cell synapses can be readily distinguished from each other, as basket cells make en passant and terminaux synapses primarily onto postsynaptic cell bodies, whereas chandelier cells make distinctive long ‘cartridge’ synapses onto the axon initial segments of postsynaptic neurons^64,65^.

### Markers for plasticity at glutamatergic synapses

The size of both pre- and post-synaptic compartments has been correlated with synaptic strength, and so differences in the size and intensity of **Synapsin1** and **PSD95** staining could be used as measure of synaptic potentiation or deprivation as a result of experience^66–68^. Whisker deprivation in adult mice has recently been shown to increase the total density of Synapsin1 staining and postsynaptic spines in spared cortex, but only after complete unilateral whisker trimming^69^. Chessboard deprivation, which we chose to promote Hebbian circuit-level plasticity rather than the wholesale homeostatic plasticity expected from a complete removal of whisker input^19,21,70,71^, may not have such drastic effects. Indeed, several long-term *in vivo* imaging studies have shown significant dendritic spine turnover, but not wholesale changes in total spine number in particular populations of L5 pyramidal neurons^16–18,72^, implying that chessboard map plasticity may have more to do with altering specific circuit connectivity than massively altering connectivity *per se*.

Differential expression of glutamate receptor subunits is also indicative of synaptic state, and can be used to characterize synaptic strength and explore the mechanisms of experience dependent synaptic change^53,73–75^. The relative brightness of immunostaining for the different receptor subunits at individual synapses could be used to define postsynaptic receptor ratios, which are thought to be highly important for regulating Hebbian, homeostatic and meta-plasticity. In particular, the ratio of NMDARs and AMPARs can been used as a proxy for synaptic potentiation^76,77^. In addition, changes in AMPA receptor subunits **GluR1** and **GluR2** are associated with Hebbian and homeostatic synaptic plasticity^67,78–81^, while changes in expression of NMDA receptor subunits **NR2A** and **NR2B** are associated with developmental and adult metaplasticity^49,52,53,82–88^. **GluR1**, in particular, has been implicated as a key player in map plasticity driven by chessboard deprivation^89^.

We should also note that although we have frequently observed good spatial colocalization between all of these glutamate receptor subunit stains and PSD95-stained postsynaptic compartments (unpublished observations, but also see Figure 3), our analysis of antibody consistency across imaging sessions (Figure 4) reveals that the **NR2A** antibody we have used exhibits a very low signal to noise ratio. As a result, we recommend being highly cautious in interpreting this particular antibody channel.

Differential expression of the **vGluT1** and **vGluT2** isoforms has also associated with differences in the kinetics of presynaptic vesicle release as a result of their different rates of vesicle loading^90^, and expression of these two isoforms may be regulated to produce homeostatic plasticity^43^.

### Markers for plasticity at GABAergic synapses

Alterations in **GABA(A)R** concentration at GABAergic synapses have been linked to forms of inhibitory plasticity, which could be detected by changes in staining intensity of these molecules at synaptic subsets^91–97^. Shifts in inhibition of spared versus deprived whisker responses are thought to be involved in certain types of whisker map plasticity^98,99^, and chessboard deprivation, in particular, is thought to keep open cortical critical periods through an effect on PV+ intracortical inhibition^71^.

### Other synaptic markers

**Arc** is an immediate early gene product whose synaptic expression is modified by neural activity. It is thought to have a role in synaptic depression and potentially other forms of synaptic plasticity such as synaptic homeostasis^100–103^. However, the antibody we used here proved extremely noisy in the initial ribbons we stained for it, and we discontinued its use in later ribbons. Any analysis of this channel should be approached with caution.

Intrinsic transgenic **YFP** expression, in some cases amplified by an antibody against **GFP,** labels a subpopulation of layer 5 pyramidal neurons in the thy1-yfp line-H mice used in these experiments^22,23,104^. *In vivo* two-photon microscopy studies have observed increased dendritic spine turnover in the distal dendritic tufts of these neurons following chessboard deprivation similar to that performed in this experiment^16–18^. Analysis of this ATomo dataset may permit quantification of additional spine plasticity in deeper layers of the cortex. **Synaptopodin** is a protein associated with the spine apparatus, a structure observed at a subset of large dendritic spines and thought to be involved in synaptic plasticity^105,106^.

### Analysis recommendations & caveats

Given the-well known variability of immunohistochemical techniques in general^107,108^, and our own analysis of the antibodies used in this study (see Technical Validation, Figure 4, Table 10) we suggest that the most robust analyses of this data will be focused on comparisons of stains between cortical layers and columns within individual ribbons. Because these different regions of interest (ROIs) within each ribbon were necessarily stained under exactly the same conditions, such internal comparisons will be relatively immune to variability between stains, whether inherent to the antibody staining itself or as a result of slight differences in the staining procedure between different ribbons.

**Figure 6:**
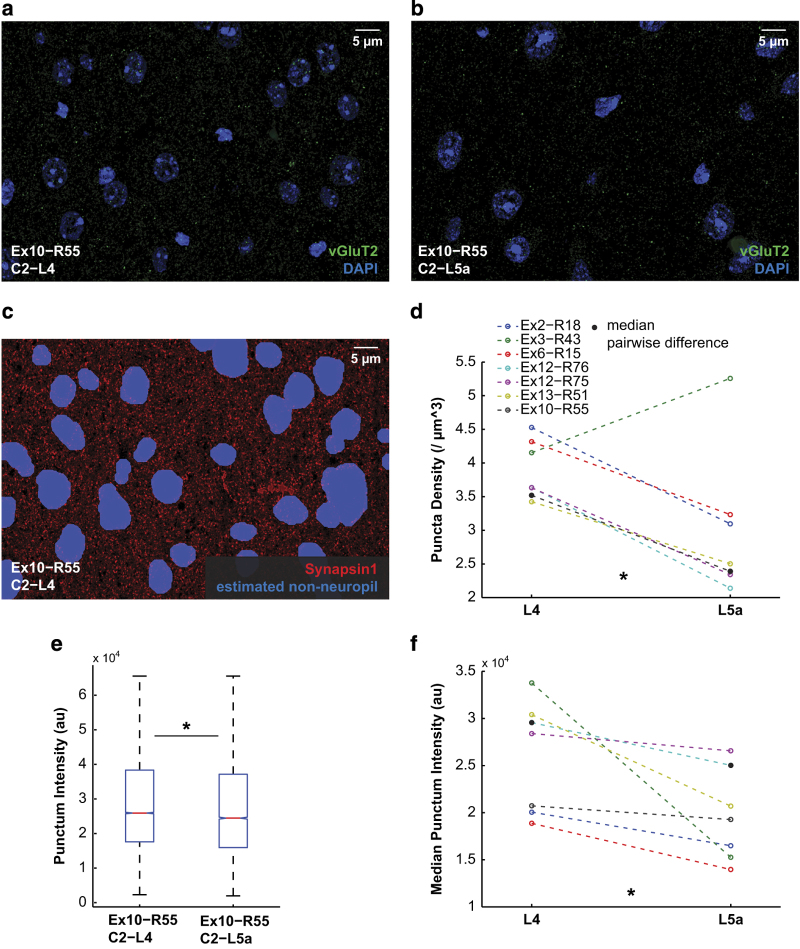
Quantification of vGluT2 staining in L4 and L5a. (**a**) Maximum intensity projection of the C2-L4 ROI of ribbon Ex10-R55 stained for vGluT2 (green) and DAPI (blue). (**b**) The same for the C2-L5a ROI. (**c**) Maximum intensity projection of the C2-L4 ROI of ribbon Ex10-R55 stained for Synapsin1 (red). Neuropil volume in each ROI is estimated by using a geometric expansion of the Synapsin1 channel’s negative space (blue), as this antibody is strongly excluded from cell bodies. The adjusted volume is used to calculate neuropil puncta densities for comparison between ROIs. (**d**) Neuropil-normalized vGluT2 puncta densities across all Chessboard Dataset data volumes for L4 ROIs (left) and L5a ROIs (right), with spared and deprived ROIs within each layer averaged together. vGluT2 density in L5a was significantly less than in L4: median difference: 46.24%; *P*=0.0313 (Wilcoxon signed-rank nonparametric test for difference of group medians); *n*=7 ribbons. (**e**) vGluT2 punctum intensity distributions for ribbon Ex10-R55 ROI C2-L4 (median 25,923 a.u.; *n*=24,189 puncta) and C2-L5a (median 24,454 a.u.; *n*=14,888 puncta). Shown are median values (red horizontal line), comparison interval (notch in box plot), interquartile range (vertical box extent), and maximum and minimum values (whiskers). Population medians are significantly different: *P*=4.85*10^−21^ (Wilcoxon signed-rank nonparametric test for difference of group medians). (**f**) Median punctum intensity across all ribbons for L4 ROIs (left) and L5a ROIs (right), with spared and deprived ROIS within each layer averaged together. Median vGluT2 punctum total intensity in L5a was significantly less than in L4: median difference: 21.56%; *P*=0.0156 (Wilcoxon signed-rank nonparametric test for difference of group medians); *n*=7 ribbons.

As a simple example of this sort of analysis, Figure 6 illustrates a quantification of differences in vGluT2 staining between L4 and L5a across ribbons. The higher density of vGluT2+ synapses in L4 is well documented^8,10,109–111^, and so we present this analysis as a simple example of how synaptic molecular differences between anatomical regions may be detected using the Chessboard Dataset.

To compare the density and intensity of staining between ROIs, we quantified individual puncta of antibody label using a 3d-Gaussian Segmentation method available in the 3d ImageJ Suite developed by Thomas Boudier and colleagues^112^. In a first pass, putative staining puncta were defined based on local maxima of staining intensity with a 3-pixel filter radius. A watershed algorithm was then used to define boundaries between these putative puncta. Within the watershed boundaries, each punctum was modeled using a 3d Gaussian filter, and segmented at a width of two standard deviations to encompass 90% of its intensity distribution. An advantage of this approach over simpler intensity threshold-based segmentation methods is that punctum size is determined by the standard deviation of the gaussian curve, and is therefore more independent of the absolute intensity of the punctum.

Within anatomically defined regions of each individual ribbon volume (Figure 2j), individual punctum fluorescence intensity and overall punctum density were calculated for each antibody stain. To avoid effects produced by differences in cell body distribution between ROIs, density measurements were calculated using estimated neuropil volume, calculated based on a dilation of the Synapsin1 channel, which is almost completely excluded from cell bodies (Figure 6c). Within individual ribbons, median punctum fluorescence intensity was compared between L4 and L5a regions using using a nonparametric Wilcoxon signed-rank test (Figure 6e). Differences in punctum density and median punctum intensity between L4 and L5a were compared across ribbons, and tested for significance using the Wilcoxon signed-rank test (Figure 6d,f).

This analysis demonstrates clearly the capacity for detecting synaptic molecular differences at the scale of the concentration of vGluT2+ thalamic inputs to L4 of barrel cortex (Figure 6). However, the same approach does not produce convincing evidence of similar wholesale differences in antibody staining between spared and deprived columns. Chessboard whisker trimming has been shown to robustly induce functional map plasticity and changes in dendritic spine density in subsets of neurons in the adult mouse^16–21,89^, but to our knowledge, no previous work has reported shifts in overall levels of synaptic proteins following chessboard trimming in the adult that approach the scale of the difference in vGluT2 expression between layers.

Indeed, the synaptic molecular changes underlying chessboard map plasticity are likely to be relatively subtle. Diverse synaptic components of the barrel cortex circuit are affected differently during map plasticity, and in many cases seem to exhibit bidirectional compensatory shifts, perhaps as a homeostatic mechanism to maintain stable overall synaptic drive^13,14,70,113–118^. As a result, analysis of wholesale differences in expression of individual synaptic proteins is likely to miss these more subtle changes occurring at specific circuit elements.

We anticipate that an important first step in analyzing these data will be to classify synaptic loci and define synaptic subtypes, which can then be analyzed for molecular changes between spared and deprived conditions. This is particularly important given the fact that many of the synaptic proteins being measured here are also expressed at non-synaptic sites^8,59,61,62,119^, which ought to be excluded from analysis of synaptic molecular changes.

We anticipate that analysis of the spatial distributions and plasticity-induced differences among these synaptic molecules will help to elucidate synaptic subsets affected by the altered sensory experience of chessboard whisker deprivation, and quantify changes in the molecular composition of these synapses in different layers of spared and deprived cortical columns. We believe some particularly promising areas of analysis include comparisons of punctum intensity ratios across synapse classes, analysis of the spatial relationships between puncta of different channels within individual synapses, and characterization of the incidence of distinct synapse classes and synapses with distinct molecular compositions onto different postsynaptic cell types.

Thus far the machine learning^40^ and other statistical approaches we have developed to define these synaptic subtypes and their molecular diversity have not proven sufficiently robust or efficient for analysis of datasets of this complexity and scale, and at any rate are well beyond the scope of the present data descriptor. We hope that others, given access to these rich data, will devise novel, creative approaches to characterizing synapses and their molecular diversity beyond what we have imagined.

## Additional information

Tables 3, 4, 5, 6, 7, 8 and 9 are only available in the online version of this paper.

**How to cite this article:** Weiler, N. C. *et al.* Synaptic molecular imaging in spared and deprived columns of mouse barrel cortex with array tomography. *Sci. Data* 1:140046 doi: 10.1038/sdata.2014.46 (2014).

## Supplementary Material



Supplementary Table 1

## Figures and Tables

**Table 1:** Animals and Ribbons.

**Animal ID**	**Dataset**	**Age**	**Sex**	**Transgenic Line**	**Imaged Ribbon(s)**	**Deprivation Pattern**	**Columns Contained** *
Ex1	Elution-Test	9 weeks	male	thy1-yfp line-H	Ribbon 2Ribbon 7Ribbon 8	C2-spared	[C1, C2]
Ex2	Chessboard	9 weeks1 day	male	thy1-yfp line-H	Ribbon 18	C2-spared	[ß, C1, C2]
Ex3	Chessboard	9 weeks1 day	male	thy1-yfp line-H	Ribbon 43	C2-spared	[C1, C2, C3]
Ex6	Chessboard	9 weeks	male	thy1-yfp line-H	Ribbon 15	C2-spared	[C1, C2]
Ex10	Chessboard	9 weeks5 days	male	thy1-yfp line-H	Ribbon 55	C2-deprived	[C1, C2]
Ex12	Chessboard	9 weeks5 days	male	thy1-yfp line-H	Ribbon 75Ribbon 76	C2-deprived	[C3, C2, C1]
Ex13	Chessboard	9 weeks2 days	male	thy1-yfp line-H	Ribbon 51	C2-spared	[C2, C1]
Ex14	Chessboard	9 weeks3 days	male	thy1-yfp line-H	Ribbon 58	C2-deprived	[C1, C2]
The nine chessboard deprived animals used in these experiments, their vital statistics, and derived ribbons/tissue samples.							

*Note that for most animals, ribbons were cut from the tissue punch starting from the E-side of the C-row (Ex1, Ex2, Ex3, Ex6, Ex10, Ex14) while others were cut from the B-side (Ex12, Ex13), producing different orientations of the columns in the final data volumes, as indicated under the ‘Columns Contained’ heading.

**Table 2:** Antibody Panel.

**Name**	**Species**	**Clonality**	**Company**	**Clone**	**Catalogue no.**	**Concent-ration**
1. Arc	Guinea Pig	poly	Synaptic Systems	N/A	156,005	1:150
2. CB	Rabbit	mono	Cell Signaling	C26D12	2,173	1:100
3. GABAARa1	Mouse	mono	NeuroMABs	N95/35	75–136	1:100
4. gActin	Mouse	mono	Sigma	2-2.1.14.17	A8481	1:100
5. GAD2	Rabbit	mono	Cell Signaling	D5G2	3,988	1:200
6. GAD6567	Rabbit	poly	AbCAM	N/A	AB11070	1:1,000
7. Gephyrin	Mouse	mono	BD Biosciences	45	612,632	1:300
8. GFP	Chicken	poly	GeneTex	N/A	GTX13970	1:100
9. GluR1	Rabbit	poly	Millipore	N/A	AB1504	1:100
10. GluR2	Mouse	mono	Millipore	6C4	MAB397	1:50
11. GluR4	Rabbit	mono	Cell Signaling	D41A11	8070P	1:50
12. NR2A	Mouse	mono	NeuroMABs	NN327/95	75–288	1:50
13. NR2B	Mouse	mono	NeuroMABs	N59/36	75–101	1:500
14. PSD95	Rabbit	mono	Cell Signaling	D27E11xP	34,505	1:200
15. PV25	Rabbit	poly	Swant	N/A	PV 25	1:100
16. Synapsin1	Rabbit	mono	Cell Signaling	D12G5	52,975	1:200
17. Synaptopodin	Rabbit	poly	Synaptic Systems	N/A	163,002	1:500
18. vGAT	Mouse	mono	Synaptic Systems	117G4	131,011	1:100
19. vGluT1	Guinea Pig	poly	Millipore	N/A	AB5905	1:5,000
20. vGluT2	Guinea Pig	poly	Millipore	N/A	AB2251	1:5,000
21. vGluT3	Guinea Pig	poly	Millipore	N/A	AB5421	1:5,000
The 21 antibodies used in the Chessboard and Elution-Test datasets (Data Citation 1) and their vital statistics. NB: some of these antibodies were used in only a subset of ribbons/data volumes. Names of antibodies used in all ribbons/data volumes are labeled in bold. (GAD2 and GAD65/67 are labeled in bold because one or the other antibody was used in every ribbon to label the same antigen.)						

**Table 3:** Chessboard Dataset Data Volumes

**Data Volume Token -->**	**Ex2R18C1**	**Ex2R18C2**	**Ex3R43C1**	**Ex3R43C2**	**Ex3R43C3**
Animal	Ex2	Ex2	Ex3	Ex3	Ex3
Ribbon	R18	R18	R43	R43	R43
Subregion	C1	C2	C1	C2	C3
					
*Size*					
x (px)	2,176	2,048	2,176	2,048	2,048
y (px)	3,328	3,200	3,328	3,200	3,328
z (px)	43	43	70	70	70
					
*Resolution*					
x (nm/px)	100	100	100	100	100
y (nm/px)	100	100	100	100	100
z (nm/px)	70	70	70	70	70
Volume (um^3)	217,976	197,263	354,845	321,126	333,971
Provenance and size of 12 data volumes derived from 8 Chessboard Dataset ribbons/tissue samples (Data Citation 1, Files 8–295 Supplementary Table 1). There are more data volumes than ribbons because the barrel columns in some ribbons were imaged without sufficient overlap for stitching to produce a combined dataset. Three ribbons (Ex2R18, Ex3R43, and Ex6R15) have separate data volumes for each barrel column, and five ribbons (Ex10R55, Ex12R75, Ex12R76, Ex13R51, and Ex14R58) have all columns included in a single data volume. The total Chessboard Dataset volume is 5,141,887 μm^3, and the average volume per ribbon (including multiple data volumes per ribbon when applicable) is 642,736 μm^3.					

**Data Volume Token -->**	**Ex6R15C1**	**Ex6R15C2**	**Ex10R55**	**Ex12R75**	**Ex12R76**
Animal	Ex6	Ex6	Ex10	Ex12	Ex12
Ribbon	R15	R15	R55	R75	R76
Subregion	C1	C2			
					
*Size*					
x (px)	3,328	3,328	3,456	5,504	6,016
y (px)	3,584	3,712	3,456	4,864	4,992
z (px)	31	31	71	36	36
					
*Resolution*					
x (nm/px)	100	100	100	100	100
y (nm/px)	100	100	100	100	100
z (nm/px)	70	70	70	70	70
Volume (um^3)	258,828	268,072	593,614	674,641	756,803
Provenance and size of 12 data volumes derived from 8 Chessboard Dataset ribbons/tissue samples (Data Citation 1, Files 8–295 Supplementary Table 1). There are more data volumes than ribbons because the barrel columns in some ribbons were imaged without sufficient overlap for stitching to produce a combined dataset. Three ribbons (Ex2R18, Ex3R43, and Ex6R15) have separate data volumes for each barrel column, and five ribbons (Ex10R55, Ex12R75, Ex12R76, Ex13R51, and Ex14R58) have all columns included in a single data volume. The total Chessboard Dataset volume is 5,141,887 μm^3, and the average volume per ribbon (including multiple data volumes per ribbon when applicable) is 642,736 μm^3.					

**Data Volume Token -->**	**Ex13R51**	**Ex14R58**
Animal	Ex13	Ex14
Ribbon	R51	R58
		
*Subregion*		
*Size*		
x (px)	5,248	4,864
y (px)	5,888	3,456
z (px)	31	42
		
*Resolution*		
x (nm/px)	100	100
y (nm/px)	100	100
z (nm/px)	70	70
Volume (um^3)	670,535	494,214
Provenance and size of 12 data volumes derived from 8 Chessboard Dataset ribbons/tissue samples (Data Citation 1, Files 8–295 Supplementary Table 1). There are more data volumes than ribbons because the barrel columns in some ribbons were imaged without sufficient overlap for stitching to produce a combined dataset. Three ribbons (Ex2R18, Ex3R43, and Ex6R15) have separate data volumes for each barrel column, and five ribbons (Ex10R55, Ex12R75, Ex12R76, Ex13R51, and Ex14R58) have all columns included in a single data volume. The total Chessboard Dataset volume is 5,141,887 μm^3, and the average volume per ribbon (including multiple data volumes per ribbon when applicable) is 642,736 μm^3.		

**Table 4:** Chessboard Dataset Antibody Channels

**Ribbon ID > v Channel ID**	**Ex2R18**	**Ex3R43**	**Ex6R15**	**Ex10R55**
1	DAPI-1	PSD95-2	DAPI-1	Arc-5
2	DAPI-2	DAPI-7	DAPI-2	Calbindin-7
3	DAPI-3	vGluT2-7	DAPI-3	DAPI-1
4	DAPI-4	vGAT-4	DAPI-4	DAPI-2
5	DAPI-5a	GABAARa1-8	DAPI-5	DAPI-3
6	DAPI-5b	NR2B-7	DAPI-6	DAPI-4
7	DAPI-6	Arc-3	GABAARa1-6	DAPI-5
8	DAPI-7	Gephyrin-1	GAD2-3	DAPI-6
9	GABAARa1-7	PV25-1	Gephyrin-2	DAPI-7
10	GAD2-4	Synaptopodin-7	GluR1-4	DAPI-8
11	GFP-5b	GAD6567-4	GluR2-5	GABAARa1-7
12	Gephyrin-1	NR2A-4	NR2A-1	GAD2-3
13	GluR1-5a	vGluT2-1	NR2B-3	GFP-4
14	GluR2-6	DAPI-8	PSD95-1	Gephyrin-2
15	GluR4-7	NR2B-3	PV25-5	GluR1-4
16	NR2A-2	GluR2-6	Synapsin1-2	GluR2-8
17	NR2B-4	DAPI-6	Synaptopodin-6	GluR4-8
18	PSD25-2	GFP-4	YFP-1	NR2A-1
19	PV25-1	vGluT1-2	vGAT-4	NR2B-3
20	Synapsin-3	GluR4-8	vGluT1-3	PSD95-1
21	Synaptopodin-6	GluR1-6	vGluT2-2	PV25-5
22	YFP-1	DAPI-1		Synapsin1-2
23	vGAT-3	Synapsin1-3		Synaptopodin-6
24	vGluT1-3	DAPI-2		YFP-1
25	vGluT2-2	DAPI-3		vGAT-4
26		DAPI-4		vGAT-5
27				vGAT-6
28				vGluT1-3
29				vGluT2-2
List of antibody channels for each ribbon in the Chessboard Dataset (Data Citation 1, Files 8–295 Supplementary Table 1). Channel names are in the form ‘[antibody name]—[imaging round]’. The left-hand column lists channel numeric tokens, which can be used in place of channel names in the OCP hdf5 interface (See Usage Notes: OCP Data Access). NB: certain stains were repeated several times (Ex3R43 vGluT2, Ex10R55 vGAT, Ex14R58 vGluT2 & vGluT2) when initial images were judged of insufficiently high quality (See Table 5). We recommend focusing analysis on the last imaged version of these repeated antibodies, though we make all data available as repeated staining may be of interest.				

**Ribbon ID > v Channel ID**	**Ex12R75**	**Ex12R76**	**Ex13R51**	**Ex14R58**
1	DAPI-1	DAPI-1	GFP-4	Arc-7
2	DAPI-2	DAPI-2	Synapsin1-2	DAPI-1
3	DAPI-3	DAPI-3	vGluT2-2	GABAARa1-6
4	DAPI-4	DAPI-4	GluR2-5	GAD2-3
5	DAPI-5	DAPI-5	vGluT1-3	Gephyrin-2
6	DAPI-6	DAPI-6	Synaptopodin-6	GFP-5
7	GABAARa1-6	GABAARa1-6	GABAARa1-6	GluR1-4
8	GAD2-3	GAD2-3	DAPI-6	GluR2-5
9	**—**	**—**	vGAT-3	NR2A-1
10	**—**	**—**	PV25-5	NR2B-3
11	**—**	**—**	NR2B-4	PSD95-1
12	Gephyrin-2	Gephyrin-2	GluR1-4	PV25-5
13	GluR1-4	GluR1-4	GAD2-3	Synapsin1-2
14	GluR2-5	GluR2-5	DAPI-5	Synaptopodin-7
15	NR2A-1	NR2A-1	DAPI-4	vGAT-4
16	NR2B-4	NR2B-4	DAPI-3	vGluT1-4
17	PSD95-1	PSD95-1	PSD95-1	vGluT1-6
18	PV25-5	PV25-5	NR2A-1	vGluT2-2
19	Synapsin1-2	Synapsin1-2	Gephyrin-2	vGluT2-3
20	Synaptopodin-6	Synaptopodin-6	DAPI-2	YFP-1
21	YFP-1	YFP-1	DAPI-1	
22	vGAT-3	vGAT-3		
23	vGluT1-3	vGluT1-3		
24	vGluT2-2	vGluT2-2		
25				
26				
27				
28				
29				
List of antibody channels for each ribbon in the Chessboard Dataset (Data Citation 1, Files 8–295 Supplementary Table 1). Channel names are in the form ‘[antibody name]—[imaging round]’. The left-hand column lists channel numeric tokens, which can be used in place of channel names in the OCP hdf5 interface (See Usage Notes: OCP Data Access). NB: certain stains were repeated several times (Ex3R43 vGluT2, Ex10R55 vGAT, Ex14R58 vGluT2 & vGluT2) when initial images were judged of insufficiently high quality (See Table 5). We recommend focusing analysis on the last imaged version of these repeated antibodies, though we make all data available as repeated staining may be of interest.				

**Table 5:** Chessboard Dataset Imaging Log

**Ribbon**	**Imaging Session**	**Imaging Date**	**primary ab antigen**	**primary ab raised in**	**primary ab vendor**	**primary ab concentration**	**secondary ab fluorophor**	**secondary ab against**	**secondary ab raised in**	**secondary ab concentration**	**exposure (ms)**	**notes/caveats**
Ex2-R18	1	14/8/2012	Gephyrin	Ms	BD Biosciences	1:300	Alexa594	Ms	Gt	1:150	250	
			PV25	Rb	Swant	1:100	Alexa647	Rb	Gt	1:150	250	
			(YFP)	N/A	N/A	N/A	N/A	N/A	N/A	N/A	1,200	
	2	15/8/2012	PSD95	Rb	Cell Signaling	1:200	Alexa647	Rb	Gt	1:150	310	
			NR2A	Ms	NeuroMABs	1:50	Alexa594	Ms	Gt	1:150	225	
			vGluT2	GP	Millipore	1:5,000	Alexa488	GP	Gt	1:150	1,150	
	3	16/8/2012	Synapsin1	Rb	Cell Signaling	1:200	Alexa488	Rb	Gt	1:150	50	slightly messy stain
			vGAT	Ms	Synaptic Systems	1:100	Alexa594	Ms	Gt	1:150	350	
			vGluT1	GP	Millipore	1:5,000	Alexa647	GP	Gt	1:150	895	slightly messy stain
	4	17/8/2012	GAD2	Rb	Cell Signaling	1:200	Alexa488	Rb	Gt	1:150	500	significant bkgd
			NR2B	Ms	NeuroMABs	1:500	Alexa594	Ms	Gt	1:150	400	
	5	23/8/2012	GluR1	Rb	Millipore	1:100	Alexa594	Rb	Gt	1:150	400	sparse stain; significant bkgd
			GFP	Chx	Genetex	1:100	Alexa488	Chx	Gt	1:150	10	
			gActin	Ms	Sigma	1:100	Alexa647	Ms	Gt	1:150	1,935	sparse stain; somatic labeling
	6	19/3/2013	GluR2	Ms	Millipore	1:50	Alexa647	Ms	Gt	1:150	1,500	dim but ubiquitous staining
			Synaptopodin	Rb	Synaptic Systems	1:500	Alexa594	Rb	Gt	1:150	100	bright somatic labeling
	7	25/3/2013	GluR4	Rb	Cell Signaling	1:50	Alexa488	Rb	Gt	1:150	600	
			GABAARa1	Ms	NeuroMABs	1:100	Alexa594	Ms	Gt	1:150	700	
	8	28/3/2013	GABAARa1	Ms	NeuroMABs	1:100	Alexa594	Ms	Gt	1:150	700	high bkgd
			GluR4	Rb	Cell Signaling	1:50	Alexa488	Rb	Gt	1:150	750	high bkgd
Ex3-R43	1	14/6/2012	Gephyrin	Ms	BD Biosciences	1:300	Alexa594	Ms	Gt	1:150	300	
			PV25	Rb	Swant	1:100	Alexa647	Rb	Gt	1:150	400	
			vGluT2	GP	Millipore	1:5,000	Alexa488	GP	Gt	1:150	400	some YFP signal remaining (NB: restained session 7)
	2	15/6/2012	PSD95	Rb	Cell Signaling	1:200	Alexa647	Rb	Gt	1:150	600	
			vGAT	Ms	Synaptic Systems	1:100	Alexa594	Ms	Gt	1:150	297	
			vGluT1	GP	Millipore	1:5,000	Alexa488	GP	Gt	1:150	140	
	3	18/6/2012	Synapsin1	Rb	Cell Signaling	1:200	Alexa488	Rb	Gt	1:150	50	
			NR2B	Ms	NeuroMABs	1:450	Alexa594	Ms	Gt	1:150	150	bleedthrough from Arc (NB: restained session 7)
			Arc	GP	Synaptic Systems	1:150	Alexa647	GP	Gt	1:150	200	bright somatic labeling
	4	21/6/2012	GAD6567	Rb	AbCAM	1:1,000	Alexa647	Rb	Gt	1:150	300	
			NR2A	Ms	NeuroMABs	1:50	Alexa594	Ms	Gt	1:150	300	
			GFP	Chx	GeneTex	1:100	Alexa488	Chx	Gt	1:150	10	
	5	20/3/2013	GluR1	Rb	Millipore	1:100	Alexa594	Rb	Gt	1:150	N/A	Excluded: Used 2 rabbit antibodies by mistake
			Synaptopodin	Rb	Synaptic Systems	1:500	Alexa488	Ms	Gt	1:150	N/A	Excluded: Used 2 rabbit antibodies by mistake
	6	22/3/2013	GluR1	Rb	Millipore	1:100	Alexa594	Rb	Gt	1:150	600	significant bkgd; some unbound secondaries ("floaters")
			GluR2	Ms	Millipore	1:50	Alexa488	Ms	Gt	1:150	400	significant bkgd
	7	26/3/2013	NR2B	Ms	NeuroMABs	1:500	Alexa594	Ms	Gt	1:150	400	
			Synaptopodin	Rb	Synaptic Systems	1:500	Alexa647	Rb	Gt	1:150	350	
			vGluT2	GP	Millipore	1:5,000	Alexa488	GP	Gt	1:150	700	
Ex6-R15	1	20/8/2012	PSD95	Rb	Cell Signaling	1:200	Alexa647	Rb	Gt	1:150	600	
			NR2A	Ms	NeuroMABs	1:50	Alexa594	Ms	Gt	1:150	500	
			(YFP)	N/A	N/A	N/A	N/A	N/A	N/A	N/A	1,000	
	2	21/8/2012	Synapsin1	Rb	Cell Signaling	1:200	Alexa488	Rb	Gt	1:150	60	slightly messy stain
			Gephyrin	Ms	BD Biosciences	1:100	Alexa594	Ms	Gt	1:150	400	
			vGluT2	GP	Millipore	1:5,000	Alexa647	GP	Gt	1:150	2,000	dim stain
	3	22/8/2012	GAD2	Rb	Cell Signaling	1:200	Alexa488	Rb	Gt	1:150	200	
			NR2B	Ms	NeuroMABs	1:500	Alexa 594	Ms	Gt	1:150	250	
			vGluT1	GP	Millipore	1:5,000	Alexa647	GP	Gt	1:150	650	
	4	23/8/2012	GluR1	Rb	Millipore	1:100	Alexa488	Ms	Gt	1:150	450	small tissue out-of-focus bubbles, captured by extended depth of field
			vGAT	Ms	Synaptic Systems	1:100	Alexa594	Rb	Gt	1:150	300	
	5	24/8/2012	PV25	Rb	Swant	1:100	Alexa647	Rb	Gt	1:150	220	
			GluR2	Ms	Millipore	1:100	Alexa594	Ms	Gt	1:150	100	
	6	24/3/2013	Synaptopodin	Rb	Synaptic Systems	1:500	Alexa488	Rb	Gt	1:150	215	high bkgd
			GABAARa1	Ms	NeuroMABs	1:100	Alexa594	Ms	Gt	1:150	750	high bkgd
Ex10-R55	1	31/10/2012	PSD95	Rb	Cell Signaling	1:200	Alexa647	Rb	Gt	1:150	1,100	
			NR2A	Ms	NeuroMABs	1:50	Alexa594	Ms	Gt	1:150	665	
			(YFP)	N/A	N/A	N/A	N/A	N/A	N/A	N/A	1,750	
	2	2/11/2012	Synapsin1	Rb	Cell Signaling	1:200	Alexa488	Rb	Gt	1:150	115	
			Gephyrin	Ms	BD Biosciences	1:100	Alexa594	Ms	Gt	1:150	550	significant drying artifacts
			vGluT2	GP	Millipore	1:5,000	Alexa647	GP	Gt	1:150	1,750	
	3	5/11/2012	GAD2	Rb	Cell Signaling	1:200	Alexa488	Rb	Gt	1:150	400	significant bkgd
			NR2B	Ms	NeuroMaB	1:500	Alexa594	Ms	Gt	1:150	420	
			vGluT1	GP	Millipore	1:5,000	Alexa647	GP	Gt	1:150	900	
	4	6/11/2012	GluR1	Rb	Millipore	1:100	Alexa594	Rb	Gt	1:150	440	
			vGAT	Ms	Synaptic Systems	1:100	Alexa647	Ms	Gt	1:150	300	significant staining noise (NB: restained sessions 5 & 6)
			GFP	Chx	GeneTex	1:100	Alexa488	Chx	Gt	1:150	25	
	5	7/11/2012	Arc	GP	Synaptic Systems	1:150	Alexa647	GP	Gt	1:150	450	bleedthrough from 594 (PV25) channel
			vGAT	Ms	Synaptic Systems	1:100	Alexa488	Ms	Gt	1:150	500	very high bkgd (NB: restained session 6)
			PV25	Rb	Swant	1:100	Alexa594	Rb	Gt	1:150	65	significant unbound secondaries ("floaters")
	6	8/11/2012	vGAT	Ms	Synaptic Systems	1:100	Alexa594	Ms	Gt	1:150	600	
			Synaptopodin	Rb	Synaptic Systems	1:500	Alexa647	Rb	Gt	1:150	500	
	7	9/11/2012	CB	Rb	Cell Signaling	1:100	Alexa647	Rb	Gt	1:150	1,700	
			GABAARa1	Ms	NeuroMABs	1:100	Alexa594	Ms	Gt	1:150	650	
	8	27/3/2013	GluR2	Ms	Millipore	1:50	Alexa594	Ms	Gt	1:150	300	significant staining noise, unbound secondaries ("floaters")
			GluR4	Rb	Cell Signaling	1:50	ALexa647	Rb	Gt	1:150	2,000	very dim staining
Ex12-R75	1	8/5/2013	PSD95	Rb	Cell Signaling	1:200	Alexa647	Rb	Gt	1:150	900	
			NR2A	Ms	NeuroMABs	1:50	Alexa594	Ms	Gt	1:150	750	
			(YFP)	N/A	N/A	N/A	N/A	N/A	N/A	N/A	750	
	2	15/5/2013	Synapsin1	Rb	Cell Signaling	1:200	Alexa647	Rb	Gt	1:150	600	
			Gephyrin	Ms	BD Biosciences	1:100	Alexa594	Ms	Gt	1:150	400	
			vGluT2	GP	Millipore	1:5,000	Alexa488	GP	Gt	1:150	400	
	3	17/5/2013	GAD2	Rb	Cell Signaling	1:200	Alexa647	Rb	Gt	1:150	1,150	
			vGAT	Ms	Synaptic Systems	1:100	Alexa594	Ms	Gt	1:150	650	
			vGluT1	GP	Millipore	1:5,000	Alexa488	GP	Gt	1:150	450	
	4	18/5/2013	GluR1	Rb	Millipore	1:100	Alexa488	Rb	Gt	1:150	800	
			NR2B	Ms	NeuroMABs	1:500	Alexa594	Ms	Gt	1:150	350	
	5	19/5/2013	PV25	Rb	Swant	1:100	Alexa647	Rb	Gt	1:150	500	
			GluR2	Ms	Millipore	1:50	Alexa594	Ms	Gt	1:150	300	significant staining noise in first half of ribbon
	6	20/5/2013	Synaptopodin	Rb	Synaptic Systems	1:500	Alexa657	Rb	Gt	1:150	1,000	significant staining noise at end of ribbon
			GABAARa1	Ms	NeuroMABs	1:100	Alexa594	Ms	Gt	1:150	600	some unbound secondaries ("floaters")
Ex12-R76	1	5/7/2013	PSD95	Rb	Cell Signaling	1:200	Alexa647	Rb	Gt	1:150	800	
			NR2A	Ms	NeuroMABs	1:50	Alexa594	Ms	Gt	1:150	350	significant staining noise in second half of ribbon
			(YFP)	N/A	N/A	N/A	N/A	N/A	N/A	N/A	500	
	2	9/5/2013	Synapsin1	Rb	Cell Signaling	1:200	Alexa647	Rb	Gt	1:150	350	
			Gephyrin	Ms	BD Biosciences	1:100	Alexa594	Ms	Gt	1:150	250	some out-of-focus tissue bubbles, largely corrected by extended depth of field
			vGluT2	GP	Millipore	1:5,000	Alexa488	GP	Gt	1:150	500	
	3	16/5/2013	GAD2	Rb	Cell Signaling	1:200	Alexa647	Rb	Gt	1:150	1,120	
			vGAT	Ms	Synaptic Systems	1:100	Alexa594	Ms	Gt	1:150	300	
			vGluT1	GP	Millipore	1:5,000	Alexa488	GP	Gt	1:150	300	
	4	18/5/2013	GluR1	Rb	Millipore	1:100	Alexa488	Rb	Gt	1:150	650	more tissue bubbles
			NR2B	Ms	NeuroMABs	1:500	Alexa594	Ms	Gt	1:150	400	
	5	21/5/2013	PV25	Rb	Swant	1:100	Alexa647	Rb	Gt	1:150	250	large tissue bubbles
			GluR2	Ms	Millipore	1:50	Alexa594	Ms	Gt	1:150	500	
	6	23/5/2013	Synaptopodin	Rb	Synaptic Systems	1:500	Alexa657	Rb	Gt	1:150	1,250	large tissue bubbles full of unbound secondaries ("floaters")
			GABAARa1	Ms	NeuroMABs	1:100	Alexa594	Ms	Gt	1:150	450	
Ex13-R51	1	6/6/2013	PSD95	Rb	Cell Signaling	1:200	Alexa647	Rb	Gt	1:150	950	somewhat messy stain
			NR2A	Ms	NeuroMABs	1:50	Alexa594	Ms	Gt	1:150	750	messy stain, staining noise
			(YFP)	N/A	N/A	N/A	N/A	N/A	N/A	N/A	N/A	very dim—did not image (but see GFP, session 4)
	2	7/6/2013	Synapsin1	Rb	Cell Signaling	1:200	Alexa647	Rb	Gt	1:150	300	
			Gephyrin	Ms	BD Biosciences	1:100	Alexa594	Ms	Gt	1:150	400	
			vGluT2	GP	Millipore	1:5,000	Alexa488	GP	Gt	1:150	700	high bkgd
	3	11/6/2013	GAD2	Rb	Cell Signaling	1:200	Alexa488	Rb	Gt	1:150	450	
			vGAT	Ms	Synaptic Systems	1:100	Alexa647	Ms	Gt	1:150	1,500	very dim—possible bleedthrough from 594 (vGluT1)
			vGluT1	GP	Millipore	1:5,000	Alexa594	GP	Gt	1:150	200	
	4	12/6/2013	GluR1	Rb	Millipore	1:100	Alexa594	Rb	Gt	1:150	400	
			NR2B	Ms	NeuroMABs	1:500	Alexa647	Ms	Gt	1:150	1,500	
			GFP	Chx	GeneTex	1:100	Alexa488	Chx	Gt	1:150	30	
	5	13/6/2013	PV25	Rb	Swant	1:100	Alexa647	Rb	Gt	1:150	500	
			GluR2	Ms	Millipore	1:50	Alexa594	Ms	Gt	1:150	250	
	6	14/6/2013	Synaptopodin	Rb	Synaptic Systems	1:2,500	Alexa488	Rb	Gt	1:150	550	Used incorrect primary concentration, but images fine
			GABAARa1	Ms	NeuroMABs	1:100	Alexa594	Ms	Gt	1:150	800	
Ex14-R58	1	29/11/2012	NR2A	Ms	NeuroMABs	1:50	Alexa594	Ms	Gt	1:150	500	
			PSD95	Rb	Cell Signaling	1:200	Alexa647	Rb	Gt	1:150	600	
			(YFP)	N/A	N/A	N/A	N/A	N/A	N/A	N/A	1,150	
	2	30/11/2012	Synapsin1	Rb	Cell Signaling	1:200	Alexa647	Rb	Gt	1:150	200	
			Gephyrin	Ms	Millipore	1:100	Alexa594	Ms	Gt	1:150	300	mistakenly used 2 mouse primaries: imaged bright Gephyrin at low exposure (little vGluT2 visible)
			vGluT2	Ms	NeuroMABs	1:5,000	Alexa594	Ms	Gt	1:150	2,000	mistakenly used 2 mouse primaries: imaged dim vGluT2 at high exposure (incl. highly saturated Gephyrin puncta) (NB: restained vGluT2 in session 3)
	3	1/12/2012	GAD2	Rb	Cell Signaling	1:200	Alexa647	Rb	Gt	1:150	1,000	
			NR2B	Ms	NeuroMABs	1:500	Alexa594	Ms	Gt	1:150	350	
			vGluT2	GP	Millipore	1:5,000	Alexa488	GP	Gt	1:150	800	very high bkgd—possibly unusable
	4	4/12/2012	GluR1	Rb	Millipore	1:100	Alexa488	Rb	Gt	1:150	550	many unbound secondaries ("floaters")
			vGAT	Ms	Synaptic Systems	1:100	Alexa594	Ms	Gt	1:150	550	
			vGluT1	GP	Millipore	1:5,000	Alexa647	GP	Gt	1:150	1,500	significant staining noise
	5	5/12/2012	PV25	Rb	Synaptic Systems	1:100	Alexa647	Rb	Gt	1:150	300	dim DAPI; staining noise at end of ribbon
			GluR2	Ms	Millipore	1:100	Alexa594	Ms	Gt	1:150	500	
			GFP	Chx	GeneTex	1:100	Alexa488	Chx	Gt	1:150	15	staining noise at end of ribbon
	6	6/12/2012	CB	Rb	Cell Signaling	1:100	Alexa647	Rb	Gt	1:150	1,932	quite dim
			GABAARa1	Ms	NeuroMABs	1:50	Alexa488	Ms	Gt	1:150	600	bad bkgd (some GFP remaining from session 5?)
			vGluT1	GP	Millipore	1:5,000	Alexa594	GP	Gt	1:150	500	
	7	7/12/2012	Synaptopodin	Rb	Synaptic Systems	1:500	Alexa488	Rb	Gt	1:150	250	still some GFP remaining from session 5?
			gActin	Ms	Sigma	1:100	Alexa594	Ms	Gt	1:150	358	some nuclear staining; bleedthrough from Arc
			Arc	GP	Synaptic Systems	1:150	Alexa647	GP	Gt	1:150	1,035	some nuclear staining; staining noise at start of ribbon
This table contains a log of all stains/imaging sessions included in the Chessboard Dataset (Data Citation 1, Files 8–295 Supplementary Table 1). All secondary antibodies were raised in goat and applied at a concentration of 1:150.												

**Table 6:** Elution-Test Dataset Data Volumes

**Data Volume Token -->**	**Ex1R02A**	**Ex1R07A**	**Ex1R07B**	**Ex1R07C**
Animal	Ex1	Ex1	Ex1	Ex1
Ribbon	R02A	R07A	R07B	R07C
				
*Size*				
x (px)	1,388	1,388	1,388	1,388
y (px)	1,040	1,040	1,040	1,040
z (px)	4	14	6	16
				
*Resolution*				
x (nm/px)	100	100	100	100
y (nm/px)	100	100	100	100
z (nm/px)	70	70	70	70
Volume (um^3)	N/A (not contiguous sections)	14,146	6,063	16,167
Provenance and size of 7 data volumes derived from 8 Elution-Test Dataset ribbon segments/tissue samples (Data Citation 1, Files 1–7 Supplementary Table 1). In this dataset, each ribbon was divided into distinct segments, which were stained separately with different antibody panels. These ribbon segments are designated with the letters A through F. The total Elution-Test dataset volume is 88,921 μm^3, and the average data volume size is 14,820 μm^3.				

**Data Volume Token -->**	**Ex1R08D**	**Ex1R08E**	**Ex1R08F**
Animal	Ex1	Ex1	Ex1
Ribbon	R08D	R08E	R08F
			
*Size*			
x (px)	1,388	1,388	1,388
y (px)	1,040	1,040	1,040
z (px)	15	21	16
			
*Resolution*			
x (nm/px)	100	100	100
y (nm/px)	100	100	100
z (nm/px)	70	70	70
Volume (um^3)	15,157	21,220	16,167
Provenance and size of 7 data volumes derived from 8 Elution-Test Dataset ribbon segments/tissue samples (Data Citation 1, Files 1–7 Supplementary Table 1). In this dataset, each ribbon was divided into distinct segments, which were stained separately with different antibody panels. These ribbon segments are designated with the letters A through F. The total Elution-Test dataset volume is 88,921 μm^3, and the average data volume size is 14,820 μm^3.			

**Table 7:** Elution-Test Dataset Antibody Channels

**Ribbon ID > v Channel ID**	**Ex1R02A**	**Ex1R07A**	**Ex1R07B**	**Ex1R07C**
1	DAPI-1	DAPI-1	DAPI-1	DAPI-1
2	DAPI-2	DAPI-2	DAPI-2	DAPI-2
3	DAPI-3	DAPI-3	DAPI-3	DAPI-3
4	NR2B-1	NR2A-1	GAD2-1	PV25-1
5	NR2B-2	NR2A-2	GAD2-2	PV25-2
6	NR2B-3	NR2A-3	GAD2-3	PV25-3
7	Synapsin1-1	PSD95-1	vGAT-1	Gephyrin-1
8	Synapsin1-2	PSD95-2	vGAT-2	Gephyrin-2
9	Synapsin1-3	PSD95-3	vGAT-3	Gephyrin-3
10	vGluT2-1	vGluT1-1		
11	vGluT2-2	vGluT1-2		
12	vGluT2-3	vGluT1-3		
List of antibody channels for each ribbon in the Elution-Test Dataset (Data Citation 1, Files 1-7 Supplementary Table 1). Channel names are in the form ‘[antibody name]—[imaging round]’.				

**Ribbon ID > v Channel ID**	**Ex1R08D**	**Ex1R08E**	**Ex1R08F**
1	DAPI-1	DAPI-1	Arc-1
2	DAPI-2	DAPI-2	Arc-2
3	DAPI-3	DAPI-3	Arc-3
4	GluR1-1	GABAARa1-1	DAPI-1
5	GluR1-2	GABAARa1-2	DAPI-2
6	GluR1-3	GABAARa1-3	DAPI-3
7	GluR2-1	Synaptopodin-1	GluR4-1
8	GluR2-2	Synaptopodin-2	GluR4-2
9	GluR2-3	Synaptopodin-3	GluR4-3
10			
11			
12			
List of antibody channels for each ribbon in the Elution-Test Dataset (Data Citation 1, Files 1-7 Supplementary Table 1). Channel names are in the form ‘[antibody name]—[imaging round]’.			

**Table 8:** Elution-Test Dataset Imaging Log

**Ribbon**	**Imaging Session**	**Imaging Date**	**primary ab antigen**	**primary ab raised in**	**primary ab vendor**	**primary ab concentration**	**secondary ab fluorophor**	**secondary ab against**	**secondary ab raised in**	**secondary ab concentration**	**exposure (ms)**	**notes/caveats**
Ex1-R02-A	1		Synapsin1	Rb	Cell Signaling	1:300	Alexa 647	Rb	Gt	1:150	592	
			NR2B	Ms	Millipore	1:500	Alexa 594	Ms	Gt	1:150	667	
			vGluT2	GP	Millipore	1:5,000	Alexa 488	GP	Gt	1:150	788	
	2		Synapsin1	Rb	Cell Signaling	1:300	Alexa 647	Rb	Gt	1:150	592	
			NR2B	Ms	Millipore	1:500	Alexa 594	Ms	Gt	1:150	667	saturated (would now image at 274 ms)
			vGluT2	GP	Millipore	1:5,000	Alexa 488	GP	Gt	1:150	788	
	3		Synapsin1	Rb	Cell Signaling	1:300	Alexa 647	Rb	Gt	1:150	592	somewhat saturated (would now image at 459 ms)
			NR2B	Ms	Millipore	1:500	Alexa 594	Ms	Gt	1:150	667	saturated (would now image at 235 ms)
			vGluT2	GP	Millipore	1:5,000	Alexa 488	GP	Gt	1:150	788	somewhat saturated (would now image at 575 ms)
Ex1-R07-A	1	3/6/2014	PSD95	Rb	Cell Signaling	1:300	Alexa 647	Rb	Gt	1:150	2,050	
			NR2A	Ms	Millipore	1:50	Alexa 594	Ms	Gt	1:150	1,160	
			vGluT1	GP	Millipore	1:5,000	Alexa 488	GP	Gt	1:150	843	
	2	4/6/2014	PSD95	Rb	Cell Signaling	1:300	Alexa 647	Rb	Gt	1:150	2,050	
			NR2A	Ms	Millipore	1:50	Alexa 594	Ms	Gt	1:150	1,160	
			vGluT1	GP	Millipore	1:5,000	Alexa 488	GP	Gt	1:150	843	
	3	4/6/2014	PSD95	Rb	Cell Signaling	1:300	Alexa 647	Rb	Gt	1:150	2,050	
			NR2A	Ms	Millipore	1:50	Alexa 594	Ms	Gt	1:150	1,160	
			vGluT1	GP	Millipore	1:5,000	Alexa 488	GP	Gt	1:150	843	
Ex1-R07-B	1	3/6/2014	GAD2	Rb	Cell Signaling	1:200	Alexa 488	Rb	Gt	1:150	1,180	
			vGAT	Ms	Synaptic Systems	1:100	Alexa 594	Ms	Gt	1:150	1,090	
	2	4/6/2014	GAD2	Rb	Cell Signaling	1:200	Alexa 488	Rb	Gt	1:150	1,180	
			vGAT	Ms	Synaptic Systems	1:100	Alexa 594	Ms	Gt	1:150	1,090	vGAT very messy
	3	4/6/2014	GAD2	Rb	Cell Signaling	1:200	Alexa 488	Rb	Gt	1:150	1,180	
			vGAT	Ms	Synaptic Systems	1:100	Alexa 594	Ms	Gt	1:150	1,090	
Ex1-R07-C	1	3/6/2014	PV25	Rb	Swant	1:100	Alexa 647	Rb	Gt	1:150	876	
			Gephyrin	Ms	BD Biosciences	1:300	Alexa 594	Ms	Gt	1:150	964	
	2	4/6/2014	PV25	Rb	Swant	1:100	Alexa 647	Rb	Gt	1:150	876	PV ubiquitous
			Gephyrin	Ms	BD Biosciences	1:300	Alexa 594	Ms	Gt	1:150	964	
	3	4/6/2014	PV25	Rb	Swant	1:100	Alexa 647	Rb	Gt	1:150	876	
			Gephyrin	Ms	BD Biosciences	1:300	Alexa 594	Ms	Gt	1:150	964	
Ex1-R08-D	1	3/6/2014	GluR1	Rb	Millipore	1:100	Alexa 488	Rb	Gt	1:150	800	background
			GluR2	Ms	Millipore	1:50	Alexa 594	Ms	Gt	1:150	761	
	2	4/6/2014	GluR1	Rb	Millipore	1:100	Alexa 488	Rb	Gt	1:150	800	
			GluR2	Ms	Millipore	1:50	Alexa 594	Ms	Gt	1:150	761	
	3	4/6/2014	GluR1	Rb	Millipore	1:100	Alexa 488	Rb	Gt	1:150	800	
			GluR2	Ms	Millipore	1:50	Alexa 594	Ms	Gt	1:150	761	
Ex1-R08-E	1	3/6/2014	Synaptopodin	Rb	Synaptic Systems	1:500	Alexa 647	Rb	Gt	1:150	1,430	
			GABAARa1	Ms	NeuroMABs	1:100	Alexa 594	Ms	Gt	1:150	2,240	almost no signal
	2	4/6/2014	Synaptopodin	Rb	Synaptic Systems	1:500	Alexa 647	Rb	Gt	1:150	1,430	
			GABAARa1	Ms	NeuroMABs	1:100	Alexa 594	Ms	Gt	1:150	2,240	
	3	4/6/2014	Synaptopodin	Rb	Synaptic Systems	1:500	Alexa 647	Rb	Gt	1:150	1,430	
			GABAARa1	Ms	NeuroMABs	1:100	Alexa 594	Ms	Gt	1:150	2,240	
Ex1-R08-F	1	3/6/2014	Arc	GP	Synaptic Systems	1:150	Alexa 647	Rb	Gt	1:150	2,750	
			GluR4	Rb	Cell Signaling	1:50	Alexa 488	Ms	Gt	1:150	1,370	
	2	4/6/2014	Arc	GP	Synaptic Systems	1:150	Alexa 647	Rb	Gt	1:150	2,750	Arc very saturated
			GluR4	Rb	Cell Signaling	1:50	Alexa 488	Ms	Gt	1:150	1,370	
	3	4/6/2014	Arc	GP	Synaptic Systems	1:150	Alexa 647	Rb	Gt	1:150	2,750	
			GluR4	Rb	Cell Signaling	1:50	Alexa 488	Ms	Gt	1:150	1,370	
This table contains a log of all stains/imaging sessions included in the Elution-Test Dataset (Data Citation 1, Files 1–7 Supplementary Table 1). All secondary antibodies raised in goat and applied at a concentration of 1:150.												

**Table 9:** Antibody Colocalization

**Correlation Peak (r)**	**Synapsin1**	**vGluT1**	**vGluT2**	**PSD95**	**GluR1**	**GluR2**	**NR2A**	**NR2B**	**GAD2**	**vGAT**	**Gephyrin**	**GABAARa1**	**PV25**	**DAPI**
Synapsin1	0.394	0.127	0.025	0.033	0.017	0.024	0.000	0.013	0.050	0.018	0.002	0.007	−0.003	−0.012
vGluT1	0.127	0.394	0.026	0.020	0.001	0.016	0.002	0.025	0.033	0.000	0.000	0.004	0.005	0.001
vGluT2	0.025	0.026	0.394	0.011	0.000	0.006	0.005	0.004	−0.002	0.002	0.032	0.008	0.013	0.017
PSD95	0.033	0.020	0.011	0.394	−0.002	0.079	0.011	0.043	0.003	−0.005	0.001	0.004	−0.005	−0.006
GluR1	0.017	0.001	0.000	−0.002	0.394	0.018	0.041	0.051	0.074	0.148	0.056	0.075	0.020	0.003
GluR2	0.024	0.016	0.006	0.079	0.018	0.394	0.016	0.033	0.015	0.017	0.018	0.033	0.004	0.018
NR2A	0.000	0.002	0.005	0.011	0.041	0.016	0.394	0.038	0.006	0.038	0.076	0.066	0.001	0.001
NR2B	0.013	0.025	0.004	0.043	0.051	0.033	0.038	0.394	0.010	0.036	0.042	0.066	−0.001	−0.001
GAD2	0.050	0.033	−0.002	0.003	0.074	0.015	0.006	0.010	0.394	0.079	0.018	0.031	0.009	0.005
vGAT	0.018	0.000	0.002	−0.005	0.148	0.017	0.038	0.036	0.079	0.394	0.054	0.079	0.029	0.004
Gephyrin	0.002	0.000	0.032	0.001	0.056	0.018	0.076	0.042	0.018	0.054	0.394	0.091	0.002	0.001
GABAARa1	0.007	0.004	0.008	0.004	0.075	0.033	0.066	0.066	0.031	0.079	0.091	0.394	0.014	0.013
PV25	−0.003	0.005	0.013	−0.005	0.020	0.004	0.001	−0.001	0.009	0.029	0.002	0.014	0.394	0.012
DAPI	−0.012	0.001	0.017	−0.006	0.003	0.018	0.001	−0.001	0.005	0.004	0.001	0.013	0.012	0.394
														
*Contour Area (px^2)*														
Synapsin1	5.77	9.13	8.82	19.78	9.22	14.59	125.38	15.25	8.81	9.57	16.48	13.69	318.64	156.17
vGluT1	9.12	5.69	7.63	21.81	21.50	16.59	11.57	9.49	6.96	8.37	352.89	13.80	5.12	21.95
vGluT2	8.76	7.63	5.36	9.61	388.84	10.56	11.79	10.71	30.38	131.79	7.87	12.46	9.54	36.26
PSD95	19.71	21.76	9.61	3.96	35.87	6.72	7.04	6.47	14.42	20.83	28.25	7.22	21.55	38.20
GluR1	9.17	62.60	219.17	35.79	5.07	14.35	11.85	8.70	10.32	8.11	14.38	11.88	12.06	397.91
GluR2	14.54	16.54	10.53	6.72	14.37	4.80	15.18	9.18	14.41	13.98	15.45	13.44	20.89	32.03
NR2A	82.12	10.79	11.85	7.03	11.88	15.11	2.73	11.00	24.73	11.37	8.55	12.00	15.47	67.40
NR2B	15.11	9.46	10.72	6.46	8.69	9.17	11.02	4.03	18.66	10.05	12.94	10.21	455.36	247.07
GAD2	8.77	6.93	29.41	1.75	10.34	14.33	24.84	18.63	5.31	10.64	22.54	15.21	12.65	30.15
vGAT	9.51	208.44	14.48	20.92	8.12	13.90	11.36	10.05	10.62	5.17	14.68	11.53	11.63	160.51
Gephyrin	15.86	251.98	7.86	59.59	14.38	15.40	8.53	12.91	22.43	14.65	5.00	11.68	38.20	101.67
GABAARa1	13.57	13.19	12.57	7.17	11.86	13.36	11.98	10.18	15.16	11.52	11.70	5.42	13.70	114.35
PV25	286.11	5.03	9.57	21.89	12.09	20.39	114.91	69.03	12.68	11.65	37.09	13.77	5.18	72.26
DAPI	145.74	22.59	37.12	35.12	288.63	32.59	78.27	321.33	29.96	304.59	26.77	116.32	73.49	18.00
														
*Contour Diameter (px)*														
Synapsin1	2.71	3.41	3.35	5.02	3.43	4.31	12.64	4.41	3.35	3.49	4.58	4.18	20.14	14.10
vGluT1	3.41	2.69	3.12	5.27	5.23	4.60	3.84	3.48	2.98	3.26	21.20	4.19	2.55	5.29
vGluT2	3.34	3.12	2.61	3.50	22.25	3.67	3.87	3.69	6.22	12.95	3.17	3.98	3.49	6.79
PSD95	5.01	5.26	3.50	2.25	6.76	2.92	2.99	2.87	4.28	5.15	6.00	3.03	5.24	6.97
GluR1	3.42	8.93	16.71	6.75	2.54	4.27	3.88	3.33	3.63	3.21	4.28	3.89	3.92	22.51
GluR2	4.30	4.59	3.66	2.93	4.28	2.47	4.40	3.42	4.28	4.22	4.43	4.14	5.16	6.39
NR2A	10.23	3.71	3.88	2.99	3.89	4.39	1.87	3.74	5.61	3.81	3.30	3.91	4.44	9.26
NR2B	4.39	3.47	3.70	2.87	3.33	3.42	3.75	2.27	4.87	3.58	4.06	3.60	24.08	17.74
GAD2	3.34	2.97	6.12	1.49	3.63	4.27	5.62	4.87	2.60	3.68	5.36	4.40	4.01	6.20
vGAT	3.48	16.29	4.29	5.16	3.21	4.21	3.80	3.58	3.68	2.56	4.32	3.83	3.85	14.30
Gephyrin	4.49	17.91	3.16	8.71	4.28	4.43	3.30	4.06	5.34	4.32	2.52	3.86	6.97	11.38
GABAARa1	4.16	4.10	4.00	3.02	3.89	4.12	3.91	3.60	4.39	3.83	3.86	2.63	4.18	12.07
PV25	19.09	2.53	3.49	5.28	3.92	5.10	12.10	9.38	4.02	3.85	6.87	4.19	2.57	9.59
DAPI	13.62	5.36	6.87	6.69	19.17	6.44	9.98	20.23	6.18	19.69	5.84	12.17	9.67	4.79
We performed a pairwise 2-dimensional cross-correlation analysis to quantify spatial correlations (colocalization) between the principal antibodies used in these experiments This table displays 2-d cross-correlation peak, half-max contour area, and equivalent circular diameter values for each pairwise combination of antibodies. (See Technical Validation: Antibody Specificity; Figure 3).														

**Table 10:** Staining Robustness to Strip-Stain Cycles.

**Antibody Name**	**R**	**R (rotated)**	**% Consistent**	**% Consistent (rotated)**	**Background % Consistent**	**Background % Consistent (rotated)**
Synapsin1	0.86	0.01	43.51	6.32	88.67	76.32
NR2B	0.44	−0.01	14.95	0.57	76.32	96.50
vGluT2	0.52	−0.01	10.66	1.05	96.50	95.35
PSD95	0.84	−0.01	47.51	3.83	94.11	85.37
NR2A	0.04	0.00	5.35	3.21	88.96	88.51
vGluT1	0.34	0.01	23.01	5.89	85.58	80.12
GAD2	0.62	0.03	23.29	3.13	93.93	90.98
vGAT	0.27	−0.01	15.27	4.45	82.55	78.74
PV25	0.64	−0.03	19.31	12.43	40.13	34.15
Gephyrin	0.66	−0.01	23.30	0.91	97.85	97.22
GluR1	0.23	0.00	9.69	1.18	96.45	95.80
GluR2	0.43	0.00	17.54	2.81	91.87	89.23
GABAARa1	0.26	0.00	5.02	0.29	97.35	97.09
Synaptopodin	0.50	−0.01	19.41	1.28	95.34	93.32
GluR4	0.31	−0.01	4.12	0.44	97.44	97.24
Arc	0.04	0.01	3.78	3.19	46.72	46.31
This table lists Pearson’s correlation coefficient and Percent Consistency of foreground and background pixels for imaging sessions 1 and 3 of each antibody included in the technical validation analysis shown in Figure 4. Percent Consistent is calculated as the percent of pixels classified as foreground (above threshold) in either session 1 or 3 that were so classified in both sessions. Background Percent Consistent is calculated with the same algorithm but for subthreshold pixels. Values are shown for both normally registered imaging sessions and 180° rotated imaging sessions. (See Technical Validation: Antibody Consistency; Figure 4).						
